# Advances in immunotherapy for glioblastoma multiforme

**DOI:** 10.3389/fimmu.2022.944452

**Published:** 2022-10-12

**Authors:** Ahmad Bakur Mahmoud, Reham Ajina, Sarah Aref, Manar Darwish, May Alsayb, Mustafa Taher, Shaker A. AlSharif, Anwar M. Hashem, Almohanad A. Alkayyal

**Affiliations:** ^1^ College of Applied Medical Sciences, Taibah University, Almadinah Almunwarah, Saudi Arabia; ^2^ Strategic Research and Innovation Laboratories, Taibah University, Almadinah Almunwarah, Saudi Arabia; ^3^ King Abdullah International Medical Research Centre, King Saud University for Health Sciences, Ministry of National Guard Health Affairs, Riyadh, Saudi Arabia; ^4^ King Fahad Hospital, Ministry of Health, Almadinah Almunwarah, Saudi Arabia; ^5^ Vaccines and Immunotherapy Unit, King Fahd Medical Research Center; King Abdulaziz University, Jeddah, Saudi Arabia; ^6^ Department of Medical Microbiology and Parasitology, Faculty of Medicine, King Abdulaziz University, Jeddah, Saudi Arabia; ^7^ Department of Medical Laboratory Technology, University of Tabuk, Tabuk, Saudi Arabia; ^8^ Immunology Research Program, King Abdullah International Medical Research Center, Riyadh, Saudi Arabia

**Keywords:** immunotherapy, glioblastoma multiforme, glioma, checkpoint inhibitors, CAR-T cells, oncolytic virotherapy, viral vector

## Abstract

Glioblastoma multiforme (GBM) is the most common and aggressive malignant brain tumor of the central nervous system and has a very poor prognosis. The current standard of care for patients with GBM involves surgical resection, radiotherapy, and chemotherapy. Unfortunately, conventional therapies have not resulted in significant improvements in the survival outcomes of patients with GBM; therefore, the overall mortality rate remains high. Immunotherapy is a type of cancer treatment that helps the immune system to fight cancer and has shown success in different types of aggressive cancers. Recently, healthcare providers have been actively investigating various immunotherapeutic approaches to treat GBM. We reviewed the most promising immunotherapy candidates for glioblastoma that have achieved encouraging results in clinical trials, focusing on immune checkpoint inhibitors, oncolytic viruses, nonreplicating viral vectors, and chimeric antigen receptor (CAR) immunotherapies.

## 1 Introduction

Glioblastoma multiforme (GBM) is the most common aggressive primary brain cancer. Brain cancers are classified into either glioma (i.e., tumors originating from glial cells) or non-glioma. Based on the type of glial cell involved in the formation of the tumor, gliomas are further divided into three sub-classes: astrocytoma, ependymoma, and oligodendroglioma. GBM is a high-grade (stage IV) malignant astrocytoma. GBM is a very poor prognosis, but it is a rare tumor type with a global incidence of fewer than 10 cases per 100,000 individuals. GBM can arise at any age; however, it is primarily diagnosed at older ages, with a median age at diagnosis of 65 years, and it is more common in males than females (1).

**Figure 1:**
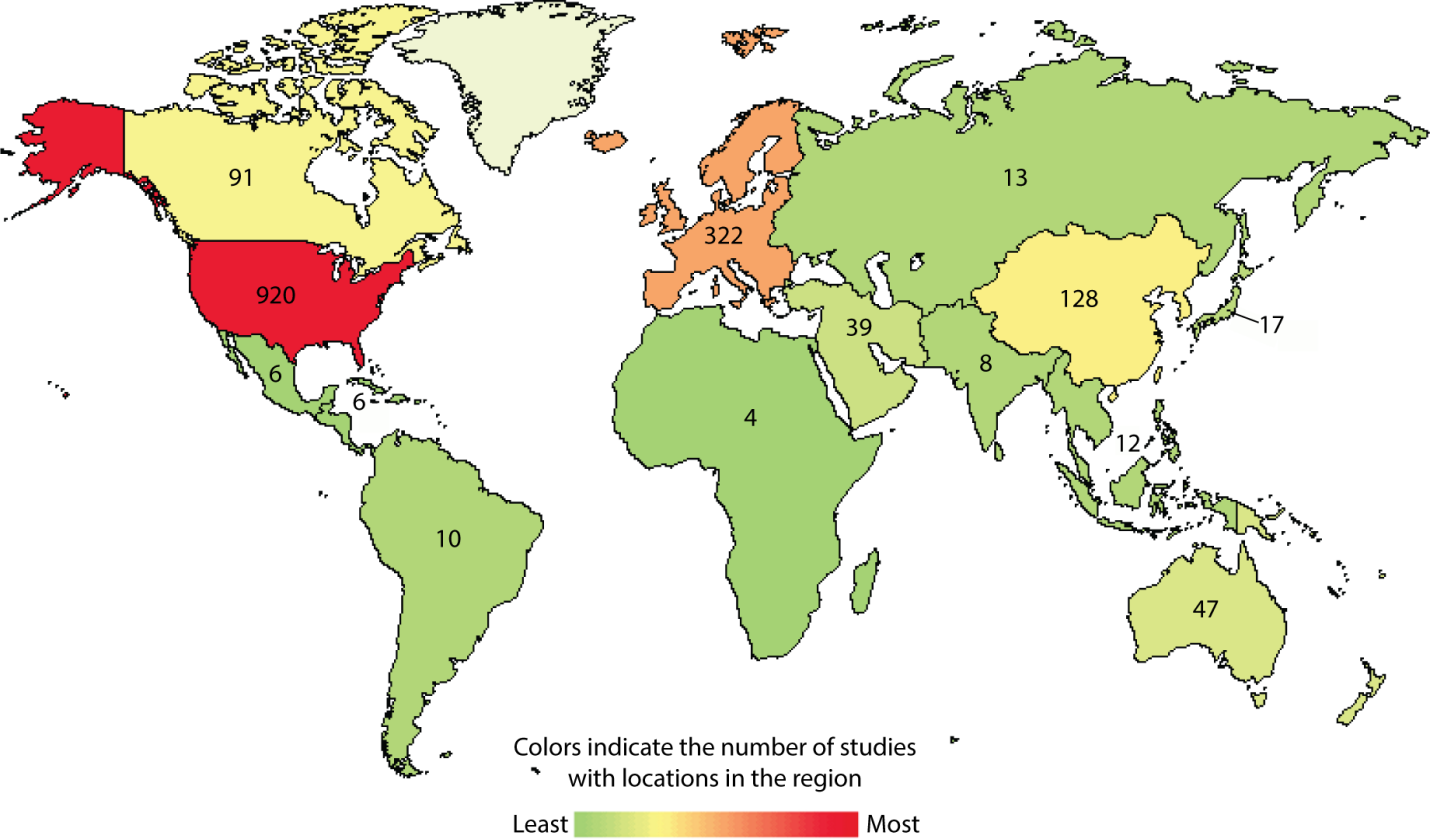
Current landscape for interventional immunotherapy clinical trials for GBM. Using clinicaltrials.gov/, condition or disease “glioblastoma”, type “interventional studies” and start study “01/01/2005” accessed on 03/20/2022.

There is an urgent need to improve the current treatment options for GBM (2, 3). The current standard of care begins with tumor debulking *via* surgical resection of the tumor, followed by concurrent radiotherapy and temozolomide (TMZ) chemotherapy. This standard therapy was suggested by the phase III EORTC 26981/22981-NCIC CE3 study in 2005 (2), and it was later supported by several other trials (3). The 5-year relative survival rate for GBM is only 7.2%, and the median survival after diagnosis is around 8 months. Almost all GBM tumors that respond to first-line therapy recur (1). This poor prognosis is not due to a lack of trials; more than 1,200 investigational clinical studies have been conducted worldwide in the last 2 decades **(** **Figure 1** **)**. This observation clearly demonstrates that the majority of GBM clinical trials have failed to produce a clinically meaningful and statistically significant survival benefit. Therefore, there is an urgent need for a more successful novel therapy for GBM.

GBM exhibits several unique characteristics that are likely responsible for its poor prognosis. The brain is protected by a monolayer that separates the central nervous system (CNS) from the peripheral blood circulation, known as the blood-brain barrier (BBB). The BBB consists of endothelial cells sealed by tight junctions that are covered by a basement membrane and surrounded by pericytes and astrocyte endfeet. While the BBB can protect the brain from harm and provide a suitable environment for brain cells to function properly, it is at least partially responsible for the therapeutic resistance exhibited by GBM (4). Second, GBM tumors are complex in nature and consist of heterogeneous cells; the identity of the GBM cell origin is still uncertain (5, 6). Moreover, the immune landscape of GBM tumors is unique and tends toward an immunosuppressive microenvironment (7, 8). Several studies have indicated that the immunosuppressive tumor microenvironment of GBM enhances the induction of tumor-associated macrophages, regulatory T cells, and immunosuppressive molecules such as PDL1, TIM-3, LAG-3, TIGIT, CD137, CD47, and CTLA4, allowing the GBM tumor to escape the antitumoral functions of T and NK cells (9, 10). Additionally, immunosuppressive cells such as regulatory T cells, myeloid-derived suppressive cells, tumor-associated macrophages, and regulatory T cells allow GMB cells to evade host tumor surveillance machinery and promote disease progression (7–10). Therefore, to improve the current standard of care for GBM, we must consider this complex brain biology and unravel the dynamic relationship that exists between GBM cells and various immunosuppressive components.

**Figure 2:**
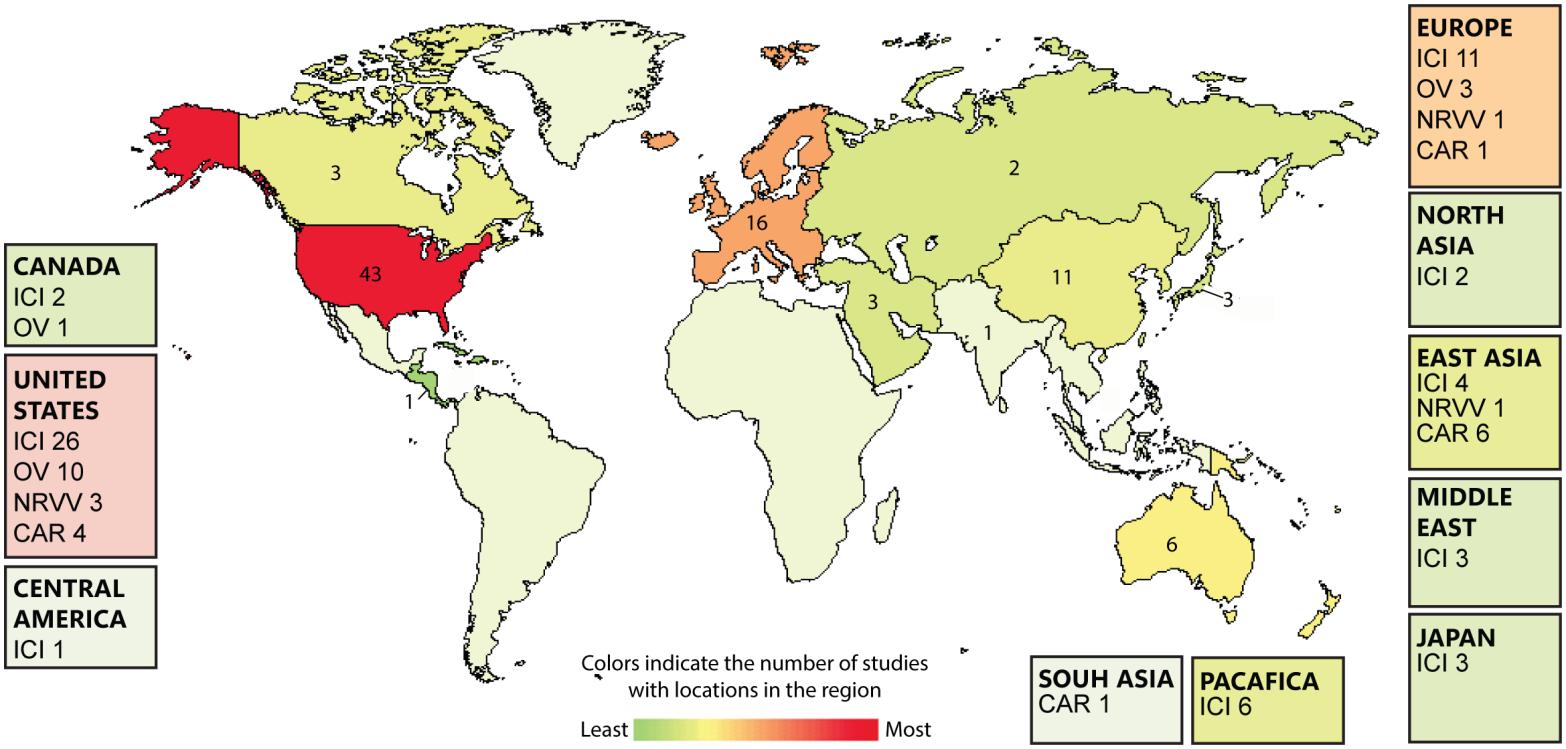
Heatmap showing the global landscape of glioblastoma (GBM) immunotherapies including; immunocheckpont inhibitors (ICI), Oncolytic virotherapy (OV), Non-replicating viral vectors (NRVV), CAR-T cell therapy (CAR). The number of GBM immunotherapies clinical trials are expressed on the country level. Data is extracted from clinicaltrials.gov/.

**Figure 3:**
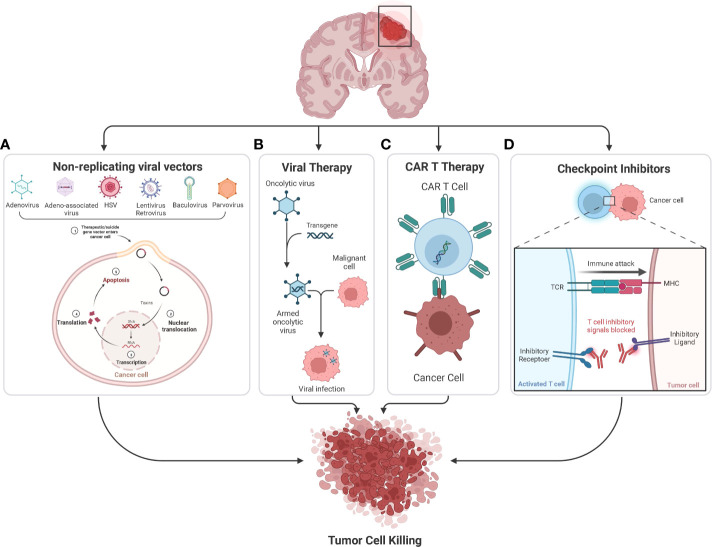
**(A)** Many nonreplicating viral vectors namely Adenovirus, Adeno-associated virus, HSV, Lentivirus, Retrovirus, Baculovirus, and Paravirus are being used to deliver suicide genes and immunostimulatory genes in the ongoing clinical studies to treat glioblastoma. The suicide gene converts nontoxic prodrugs into toxic products in tumor cells, causing tumor cell killing. **(B)** Oncolytic viruses can occur naturally or be genetically engineered by modifying natural viruses. These viruses can selectively infect and kill tumor cells without damaging the healthy cells. **(C)** CAR T-cell therapy involves genetic modification of a patient’s T-cells to produce CAR, which helps the T cells to recognize and target cancer cells. So once the modified CAR T-cells are reinfused into the patient, the new receptor will enable them to target and kill cancer cells. **(D)** Checkpoint inhibitors are monoclonal antibodies that target proteins on the surface of cancer cells or immune cells which are involved in the regulation of T and NK cell activation, key examples of which include CTLA-4 and PD-1 pathways.

Immunotherapy is promising as an effective treatment strategy for GBM. Cancer immunotherapy enhances the ability of the body’s immune system to eradicate cancer. Because immunotherapy might be successful in selectively killing cancer cells while sparing normal brain tissue (11), it is a potentially useful therapeutic strategy for GBM. There are 88 ongoing immunotherapy clinical trials for GBM **(** **Figure 2** **)**. In this review, we discuss the current clinical progress of the major types of cancer immunotherapy for the treatment of GBM, including checkpoint inhibitors, chimeric antigen receptor (CAR)-T cell therapy, oncolytic virotherapy, and non-replicating viral vectors **(** **Figure 3** **)**.

## 2 Immune checkpoint inhibitors for the treatment of glioblastoma

One of the awe-inspiring properties of the immune system is its ability to maintain a balance between T-cell activation and T-cell suppression. While effective T-cell immunity is required to protect the body from infections and cancer, a persistent T-cell response can lead to chronic inflammation and tissue damage. One of the crucial mechanisms that the immune system possesses to achieve this balance is inducing the expression of co-inhibitory signals, also known as immune checkpoints. Immune checkpoints are surface molecules that, when bound to their counter ligands or receptors, regulate host immunity. Cancer cells tend to employ this mechanism to evade T-cell immune-mediated destruction. This finding led to the development of immune checkpoint-targeting therapies that aimed to restore T-cell anti-tumor immunity (12–14).

Immune checkpoint blockade has attracted significant attention in the last decade due to its success in treating a wide range of malignancies. This success was highlighted in the magazine Science, where cancer immunotherapy was declared the 2013 “Breakthrough of the Year”. Later, the 2018 Nobel Prize in Physiology or Medicine was awarded to James P. Allison and Tasuku Honjo for their discovery of immune checkpoint inhibitors. However, unfortunately, immune checkpoint blockade has had little therapeutic success in GBM. Therefore, combination therapy of checkpoint inhibitors with other immune-stimulating agents is the focus of many ongoing clinical trials **(** **Supplementary Table 1** **)**. In this section, we describe the major immune checkpoints that have been targeted in clinical GBM studies.

### 2.1 Anti-PD-1/PD-L1

Programmed death 1 (PD-1, CD279) is a co-inhibitory molecule expressed predominantly on the surface of activated T cells. When PD-1 binds to its ligand (PD-L1, CD274) on the surface of tumor cells or antigen-presenting cells (APCs), it induces T-cell apoptosis and anergy (15, 16). Furthermore, PD-1 has been shown to stimulate regulatory T-cell (T_reg_) proliferation and decrease natural killer (NK) cell and B-cell responses (17). Focusing on glioblastoma, PD-L1 in GBM tumors is detectable in most patients; however, the percentage of PD-L1-positive cells varies among patients. For example, Nduom EK et al. reported that 61% of the tumor tissues they evaluated expressed PD-L1 (17). Studies have shown that PD-L1 expression in GBM tumor cells corresponds to levels of malignancy and tumor aggressiveness, which could enhance the risk of immune evasion and serve as a prognostic predictor (18–20). Over the last few years, several clinical trials have examined PD-1 and PD-L1 inhibitors for treating GBM **(** **Supplementary Table 1** **)**. Preliminary clinical reports of anti-PD-1/PD-L1 as monotherapy demonstrated limited efficacy in patients with GBM compared with control groups (21). The immunosuppressive properties of GBM could have hindered the efficacy of various anti-PD-L1/PD-1 treatments. Three phase III clinical trials were conducted by Bristol Myers Squibb to evaluate the efficacy of nivolumab, a checkpoint inhibitor targeting PD-1. The open-label, randomized CheckMate-143 trial was the first phase III study examining the effect of nivolumab in GBM patients. In this clinical trial, the efficacy of nivolumab vs. bevacizumab (anti-vascular endothelial growth factor [VEGF] antibody) was evaluated. However, the results were disappointing due to the lack of improvement in median overall survival (mOS) between the two treatment groups (22). The CheckMate-498 trial (NCT02617589) evaluated the effect of nivolumab with radiotherapy compared to TMZ and radiotherapy in newly diagnosed ​​O6-methylguanine-DNA methyltransferase (MGMT)-unmethylated GBM patients after surgical tumor resection. The CheckMate-548 trial (NCT02667587) evaluated the addition of nivolumab to the current standard of care (TMZ and radiation therapy) versus placebo plus the standard of care in patients with newly diagnosed MGMT-methylated GBM following surgical resection of the tumor​​. Unfortunately, both the CheckMate-498 and CheckMate-548 trials failed to meet their primary endpoints of overall survival at final analysis. Nonetheless, anti-PD-1 is currently approved for the treatment of solid tumors with microsatellite instability-high (MSI-H), mismatch repair deficiency (dMMR), or tumor mutation burden-high (TMB-H), including GBM. Although these cases are very rare (23), this observation highlights the potential success of immune checkpoint blockade in combination with immunotherapeutic agents that induce tumor immune sensitivity.

### 2.2 Anti-CTLA-4

The cytotoxic T-lymphocyte-associated antigen 4 (CTLA-4, CD152​​) is a co-inhibitory molecule that binds to the cell surface ligands CD80 (B7-1) and CD86 (B7-2) on the APC with the highest avidity, outcompeting CD28 binding. CD28 is a co-stimulatory molecule that is also expressed on T cells, and it binds to the same ligands as CTLA-4 (24). Because CTLA-4 can interrupt CD28 co-stimulation signaling, it can suppress T-cell antigen-specific responses and is thus considered one of the central negative regulators of T-cell activation (25). Expression of CTLA-4 has been shown to be higher in high-grade gliomas compared with low-grade gliomas, indicating that the expression of CTLA-4 is positively correlated with cancer severity (26). Currently, a phase II clinical study on anti-CTLA-4 is examining TMZ treatment alone versus TMZ with ipilimumab (anti-CTLA-4) in GBM patients after radiation and chemotherapy. The lack of potential biomarkers that could determine the clinical response to anti-CTLA-4 therapy is considered a limitation of this study (27). Furthermore, a randomized phase II/III open-label study (NCT04396860) is currently ongoing to determine the efficacy of ipilimumab and nivolumab versus TMZ in patients with newly diagnosed MGMT-unmethylated GBM. Several other ongoing clinical trials are evaluating the combination efficacy of CTLA-4 with PD-1 in treating GBM to further unleash the potential of anti-CTLA-4 therapy **(** **Supplementary Table 1** **)**.

### 2.3 Anti-TIM-3

Another promising immune checkpoint inhibitor is the T-cell immunoglobulin and mucin-domain containing-3 (TIM-3). TIM-3 has multiple ligands that can be expressed on tumor cells and APCs, such as C-type lectin, galectin-9, phosphatidylserine (PtdSer), high-mobility group protein 1 (HMGB1), and carcinoembryonic antigen-related cell adhesion molecule 1 (CEACAM1) (28). TIM-3 is a co-inhibitory molecule expressed on several immune cells, including T cells, B cells, NK cells, dendritic cells (DCs), monocytes, and macrophages (29). TIM-3 is often co-expressed with PD-1 in exhausted CD8^+^ T cells (30, 31). Previous studies have shown that TIM-3 upregulation in patients with non-small cell lung carcinoma (NSCLC) could induce resistance to PD-1 blockade, suggesting that TIM-3 inhibitors might improve resistance to anti-PD-1/PD-L1 treatment (30, 32). In line with these findings, intratumoral TIM-3 expression in CD4^+^ and CD8^+^ T cells has been shown to be higher in GBM compared with low-grade gliomas, suggesting a role for TIM-3 expression in glioma severity (33). Kim et al. examined the therapeutic effect of TIM-3 in different setups (8 arms) in a murine glioma model: control, stereotactic radiosurgery (SRS), anti-PD-1, anti-TIM-3, anti-PD-1 + SRS, anti-TIM-3 + SRS, anti-PD-1 + anti-TIM-3, and triple therapy with anti-PD-1 + anti-TIM-3 + SRS. When compared with the other arms, the triple therapy demonstrated complete long-term survival and an increase in immune cell infiltration, immune cell activity, and memory. These results highlight the therapeutic potential of this novel triple combination in GBM (34). Currently, anti-TIM-3 therapy is being explored in clinical settings for several cancer indications, including GBM (NCT03961971).

### 2.4 Anti-LAG-3

Lymphocyte-activation gene 3 (LAG-3) is a co-inhibitory molecule expressed on several immune cells, including CD4^+^ and CD8^+^ T cells, plasmacytoid DCs (pDCs), NK cells, and B cells (35–38). LAG-3 protein structure is highly similar to that of CD4, and it can thus bind to major histocompatibility complex class II (MHC-II) with high affinity. This similarity might explain why LAG-3 signaling can inhibit T-cell activation and proliferation. Not surprisingly, cancer cells tend to escape T cell-mediated immune surveillance by activating LAG-3 signaling (39). Harris-Bookman et al. demonstrated that anti-LAG-3 monotherapy or in combination with anti-PD-1 could eradicate GBM in mice. The study also demonstrated that LAG-3 was an early marker of exhausted T cells, indicating the potential therapeutic benefit of early treatment with LAG-3 antagonists (40). Currently, anti-LAG-3 or anti-CD137 alone or in combination with anti-PD1 is being investigated in a phase I trial for patients with recurrent GBM (NCT02658981).

### 2.5 Anti-TIGIT

T-cell immunoreceptor with Ig and ITIM domain (TIGIT) is a co-inhibitory molecule expressed exclusively in lymphocytes, mainly T cells and NK cells (41). Several pre-clinical studies have demonstrated that anti-TIGIT antibody can directly inhibit T-cell proliferation and improve the anti-tumor immune response as a monotherapy or in combination with PD-1 and TIM-3 inhibitors (42–44). The expression of TIGIT in CD8^+^ T cells at the tumor site in GBM patients was found to be upregulated compared with healthy individuals (50% vs. 14%, respectively) (45). In line with these findings, Hung et al. reported that the expression of the TIGIT ligand poliovirus receptor (PVR) was associated with poor survival in patients with glioma. This study also demonstrated that the combination of anti-TIGIT with anti-PD-1 could improve the survival rate compared with monotherapy in a murine GBM model. This observation was correlated with an increase in effector T-cell activity and downregulation of regulatory T cells (46). Therefore, TIGIT presents a promising target for immunotherapy in patients with GBM. Anti-TIGIT therapy is currently in phase I clinical development in a multicenter trial in combination with anti-PD-1 antibody for recurrent GBM (NCT04656535).

### 2.6 Anti-CD137

CD137, also known as 4-1BB, is a member of the tumor necrosis factor receptor (TNFR) family. It is a co-stimulatory molecule involved in the regulation of immune cell activation, including activation of CD4^+^ T cells, CD8^+^ T cells, NK cells, and DCs (47–50). CD137 can mediate and enhance the cytotoxic function of CD8^+^ T cells, and it signals through engagement with its ligand, 4-1BB-L, only when the T-cell receptor (TCR) signaling is strong, providing a co-stimulatory signal to T cells independently of CD28. This signaling leads to the preferential expansion of CD8^+^ T cells over CD4^+^ T cells, increasing their survival, cytotoxicity, and interferon gamma (IFNγ) production (51). Using an *in vitro* model of human glioma, IFNγ production was shown to be induced in the context of peripheral blood mononuclear cells (PBMCs) primed with tumor lysate-pulsed DCs in the presence of anti-CD137. Furthermore, PBMC cytotoxicity was enhanced when incubated with anti-CD137 antibody. The cell cytotoxicity was mediated mainly by CD4^+^ and CD8^+^ T cells (52). This observation was supported by an *in vivo* study that showed that anti-CD137 could enhance anti-tumor efficacy in glioma tumor models (53). Based on these promising results, anti-CD137 is currently being evaluated as a monotherapy in a phase I clinical trial to treat patients with recurrent GBM (NCT02658981).

While several immune checkpoint inhibitors are showing promising preclinical studies in glioma model, efficacy in clinical trials outcome is still limited, indicating that Gliomas is highly resistant to immune checkpoint therapy. Several resistance mechanisms have been suggested, such as the low number of infiltrated T cells in TME, chemotherapy mediated immunosuppression, immunosuppressive myeloid cells and the upregulation of other immune checkpoints that can block the therapy (e.g. TIM3) (54). Thus, future studies should further investigate the specific TME in Gliomas, in order to create a combinational therapy that can overcome the resistance mechanism and generate a better therapeutic outcome in GBM patients.

## 3 Oncolytic viruses for the treatment of glioblastoma

Gene therapy is an innovative treatment modality that has attracted attention over the past 3 decades as a promising therapeutic strategy for several diseases, including cancers. The brain is a critical organ, and the recent success and advancement of gene therapy treatment for retinal and brain metabolic disorders has promoted gene therapy development for malignant glioma. Clinical trials have presented compelling evidence that gene therapy is safe and efficacious, with significant efforts in translational, preclinical, and clinical development (55, 56). Gene therapy-based treatments include oncolytic virotherapy, non-replicating viral vectors, and CAR-T cell therapy. Each of these therapeutic strategies is discussed in the following sections.

A wide range of wildtype and genetically-modified viruses are being investigated as potential oncolytic agents for the treatment of GBM. Oncolytic viruses (OVs) are weakly pathogenic viruses that can selectively infect, replicate in, and kill cancer cells, leaving normal cells intact. OVs function by inducing immunogenic cancer cell death and turning the tumor microenvironment (TME) from immunosuppressive (i.e., “cold tumors”) to “hot tumors” (57). Encouraging results from preclinical studies have paved the way for the transition of several viruses into the clinical setting. **Supplementary Tables 2** and **3** summarize the completed and active clinical trials of OVs for the treatment of GBM, either as single agents or in combination with other treatment modalities. Currently at the frontline is G47Δ, a genetically modified herpes simplex OV which has recently received approval for the treatment of GBM in Japan. Perhaps the most remarkable results were those observed in Early-stage clinical trials in patients with glioblastoma using DNX-2401, PVSPIRO, and Toca 511, which have demonstrated complete durable responses in approximately 20% of patients receiving the virus intratumorally. These viruses have since been granted “Fast Track” designations by the United States Food and Drug Administration (U.S. FDA) for expedited drug review (58).

### 3.1 Adenovirus

Adenovirus (Ad) is a double-stranded DNA, non-enveloped virus with an icosahedral capsid. It is one of the most frequently employed oncolytic viruses; at least three oncolytic Ads have been generated for the treatment of GBM.

#### 3.1.1 DNX-2401 (tasadenoturev, delta-24-RGD)

DNX-2401 is a recombinant Ad serotype 5 genetically engineered through a 24-bp deletion in the retinoblastoma (Rb)-binding domain of the E1A gene, rendering it selective for Rb-deficient tumor cells 8. Glioma cells are known to express low levels of the coxsackie-Ad receptor on their surface (59). In an attempt to overcome this and to increase viral tumor selectivity, an integrin-binding RGD-4C peptide motif was inserted into the adenoviral fiber, enabling its interaction with integrins αvβ3 and αvβ5, which are commonly overexpressed on the surface of GBM cells (60). Preclinical studies have demonstrated DNX-2401 to be efficacious in glioma xenograft mouse models receiving intratumoral injections of the virus by direct oncolysis in addition to eliciting anti-tumor immune responses (61, 62). These observations provided proof-of-concept for the translational assessment of DNX-2401 in a dose-escalating phase I clinical trial (NCT00805376). In the trial, 37 patients with recurrent malignant glioma were enrolled in two groups, with patients in group A (*n*=25) receiving an intratumoral injection of the virus through a biopsy needle into the tumor to evaluate safety, and patients in group B (*n*=11) receiving an intratumoral injection through an implanted catheter, followed 14 days later by tumor resection to assess the mechanism of action. In group A, 72% (18/25) of patients exhibited tumor reduction. The median overall survival was 9.5 months, and 20% (5/25) of the patients survived for more than 36 months after the initial treatment. The median overall survival time in group B was 13 months, and two patients survived for 24 months. Immunohistochemical evaluation of post-treatment specimens revealed CD8^+^ and T-bet^+^ cell infiltration, suggesting the production of a Th1 cell-mediated immune response. Interestingly, expression of PD-1 and PD-L1 were not affected by the treatment; in contrast, the expression of TIM-3 was reduced in response to therapy (63). A subsequent phase III trial to further explore DNX-2401 as a monotherapy for GBM patients is being planned by DNAtrix.

Preclinical data supported the synergy of DNX-2401 with TMZ and IFNγ (64, 65). However, reports from a randomized phase Ib trial (NCT02197169; TARGET-I) suggested that the addition of IFNγ did not appear to provide additional benefit or improve survival rates compared to treatment with DNX-2401 alone (66). In contrast, reports from the phase I trial investigating the combination of DNX-2401 and TMZ in 31 patients at first recurrence of GBM demonstrated that the safety endpoint of the trial was achieved, with no virus-related toxicities or adverse events. Interestingly, studies from this trial highlighted fibroblast growth factor 2 (FGF2) as a potential prognostic marker; elevated FGF2 expression was correlated with significantly longer overall survival (64). Another trial utilizing DNX-2401 is a phase I/II trial in the Netherlands is testing the virus in patients with recurrent GBM. Although efficacy results of this trial have not yet been published, DNX-2401 treatment was shown to increase cytokine levels in CSF samples of patients, which is suggestive of the development of a pro-inflammatory microenvironment (67).

Improving GBM immunogenicity with combination immunotherapy could be an effective approach for the treatment of GBM. In the clinical setting, a phase II trial (NCT02798496: CAPTIVE/KEYNOTE-192) evaluating the combination of DNX-2401 with the anti-PD-1 antibody pembrolizumab in patients with recurrent GBM or gliosarcoma is currently ongoing. DNX-2401 is intratumorally delivered followed by intravenous administration of pembrolizumab every 3 weeks for up to 2 years or until confirmed disease progression. Interim data from 42 patients showed a median overall survival of 12.3 months, which was favorable compared with the survival observed for standard-of-care agents lomustine and temozolomide, which had a median overall survival of 7.2 months at the time. Four patients survived for more than 23 months, and 11.9% (5/42) had durable responses. No dose-limiting toxicities were observed, and adverse events were mild to moderate and unrelated to DNX-2401.

#### 3.1.2 DNX-2440

In a different approach aimed at enhancing the virus-mediated immune response, DNX-2440 was developed by modifying DNX-2401 to express the immune co-stimulator OX40 ligand (OX40L). This modification resulted in enhanced CD8^+^ T-cell proliferation and a prolonged survival rate in glioma mouse models compared with the unmodified DNX-24011 (68). DNX-2440 is currently being tested in a phase I clinical trial in patients with newly diagnosed GBM (NCT03714334).

#### 3.1.3 Onyx-015

Onyx-015 is a chimeric type 2/5 Ad with a deletion in the E1B gene that was thought to restrict viral replication to cells with a defective protein 53 (p53) pathway (69). Subsequent studies demonstrated that Onyx-015 replicated in cancer cells with wildtype p53, and some tumor cells did not support the replication of Onyx-015, suggesting a p53-independent mechanism (70–72). Onyx-015 was approved in China in 2015 for the treatment of head and neck cancers (73). Safety of administering up to 1010 plaque-forming units (pfu) of the virus at the time of tumor resection was demonstrated in a phase I clinical trial with 24 patients with malignant glioma. However, no definite anti-tumor efficacy was demonstrated in this trial, with a median progression-free survival of 46 days (74).

### 3.2 Herpesvirus

Herpes simplex virus type 1 (HSV-1) is a double-stranded DNA enveloped virus that naturally infects human neural tissues, making it an attractive candidate for GBM oncolytic virotherapy. HSV-1 represents one of the most extensively studied OV platforms; a number of recombinant oncolytic HSVs (oHSVs) attenuated to varying degrees have been generated for the treatment of GBM (75), six of which have progressed to clinical trials **(** **Supplementary Tables 2**, **3** **)**.

#### 3.2.1 HSV-1716

A first-generation oHSV, HSV-1716, harbors a deletion of 759 bases in both copies of the γ34.5 gene, the major determinant of the neurovirulence of HSV-1 (76). In principle, safety was achieved at the expense of virulence.

In normal cells, HSV-1 infection induces protein kinase R (PKR) upregulation, which in turn leads to the phosphorylation and deactivation of eukaryotic initiation factor 2 alpha (eIF2α), resulting in inhibition of viral protein translation and limiting of viral replication in the infected cell. The γ34.5 gene product binds to protein phosphatase 1 alpha (PP1α), dephosphorylating eIF2α and permitting viral protein synthesis (77). Therefore, replication of oHSVs with mutations in both copies of the γ34.5 gene is restricted to cancer cells due to their defective anti-viral PKR response (78). HSV-1716 has been shown to possess oncolytic activity *in vitro* and *in vivo*, without replicating in normal tissues (79, 80). The safety and tolerability of HSV-1716 was tested in three completed phase I clinical trials by a group in the United Kingdom (UK) who determined the maximum tolerated dose of HSV-1716 to be 10^5^ pfu (81–83). A phase I clinical trial with two pediatric patients was terminated in 2016 due to lack of recruitment and the results were not posted (NCT02031965).

#### 3.2.2 HSV-G207

The second-generation oHSV, HSV-G207, is a double mutant with deletions in both copies of the γ34.5 gene in addition to an inactivating insertion of the *Escherichia coli* lacZ reporter gene into the UL39 gene encoding infected cell protein 6 (ICP6) (84). ICP6 is the large subunit of ribonucleotide reductase, an essential enzyme for nucleotide metabolism and viral DNA synthesis in non-dividing cells (85). This deletion renders G207 specific to dividing tumor cells (84). HSV-G207 demonstrated oncolytic activity in human glioma cells and prolonged the survival of glioma xenograft models (84, 86, 87). The safety of G207 was demonstrated in a phase I clinical trial (NCT00157703) where nine patients with recurrent malignant glioma received intratumoral injections of G207 into five sites following tumor biopsy. A single dose of 5 Gy radiation was administered within the following 24 hours. Partial response was observed in six out of nine patients, and radiographic response was observed in three patients. The median overall survival was 7.5 months. The treatment was well tolerated, without incidence of HSV-related encephalitis (88). In a phase Ib/II trial, (NCT00028158), 21 patients with recurrent glioma received intratumoral injections of G207. Four patients survived for a mean 12.8 months post-inoculation compared with a mean survival of 6.2 months for the remaining 17 patients (89). In general, treatment with G207 was safe when injected at a maximum dose of 3 × 109 pfu. There are currently two ongoing phase I clinical trials evaluating the treatment in pediatric patients (NCT02457845 and NCT03911388).

#### 3.2.3 G47Δ

HSV-1 infection results in the downregulation of MHC class I expression on the surface of infected cells. To overcome this downregulation, the G207 derivative virus, G47Δ, was engineered with an additional deletion in the α47 gene and the overlapping herpes unique short 11 (US11) promoter region. The deletion of α47 increased MHC I antigen presentation and tumor infiltrating lymphocytes in G47Δ-infected human melanoma cells. In glioma xenograft mouse models, G47Δ exhibited superior viral growth and tumor lysis compared with G207 (90). Furthermore, deletion of α47 restored the replication of oHSV in GBM stem cells (GSCs), a feature that is compromised in viruses with γ34.5 deletions (91). This is particularly advantageous, as GSCs are often associated with resistance to traditional therapies (92, 93). The safety of G47Δ was tested in a phase I/II clinical trial in patients with GBM in Japan (UMIN000002661), and a subsequent phase II trial (UMIN000015995) was recently completed with results yet to be published (94). A subsequent Phase II trial (UMIN000015995) to test the efficacy of G47Δ in patients with residual of recurrent GBM has been recently completed and demonstrated that the 1-year survival rate of 13 patients reached 84.2% and an overall survival of 20.2 months after G47Δ initiation. Biopsies showed increased infiltration of CD4+ and CD8+ lymphocytes (95). Based on these results, G47 (Delytact/Teserpaturev) received conditional and time-limited approval from Japan’s Ministry of Health, Labour and Welfare (MHLW) in June 2021 for the treatment of malignant gliomas in Japan (96).

#### 3.2.4 RQNestin34.5v2

An alternative strategy to compensating for the limited replication of attenuated oHSVs lacking γ34.5 was to engineer a virus that conditionally expressed one copy of γ34.5 under the control of the nestin promoter/enhancer element. Nestin, an intermediate filament, is overexpressed in many cancers, including GBM. rQNestin34.5 has been shown to increase survival in animal models of glioma (97). rQNestin34.5v2, with an additional deletion of a fusion transcript encoding green fluorescent protein (GFP) linked to the carboxyl terminus of the ICP6 gene, is currently being investigated in a phase I clinical trial with 108 enrolled glioma patients (NCT03152318). In group A, a single dose is intratumorally injected at increasing doses until the maximum-tolerated dose is reached. Patients are then treated with cyclophosphamide (CPA) 2 days prior to an intratumoral dose of rQNestin (98, 99).

#### 3.2.5 C134

C134 is a chimeric virus lacking both copies of the γ34.5 gene and expressing the insulin receptor substrate 1 (IRS1) gene of a distantly related herpesvirus, human cytomegalovirus (HCMV) (100). IRS-1 has been demonstrated to evade PKR-mediated translational arrest and selectively restore late viral protein synthesis without neurovirulence (101). C134 exhibited superior anti-tumor effects in preclinical models of human glioma compared with γ34.5-null oHSVs (102). C134 is currently being tested in a phase I clinical trial with 24 patients with recurrent GBM (NCT03657576).

#### 3.2.6 M032

HSV-1 armed with cytokines has also been explored as a treatment for glioma (103). M032 expresses human interleukin-12 (IL-12) and has been shown to enhance the anti-tumor effect in syngeneic and xenograft mouse models (104). M032 is currently under investigation in a phase I clinical trial (NCT02062827) involving 36 patients with recurrent GBM.

### 3.3 Poliovirus

#### 3.3.1 Oncolytic polio/rhinovirus recombinant

Poliovirus is a single-stranded RNA, non-enveloped virus with an icosahedral capsid. The second oncolytic virus that received a breakthrough therapy designation for recurrent GBM from the FDA was PVSRIPO, a genetically engineered version of the live-attenuated Sabin type 1 poliovirus (PVS). PVS has natural tropism to the poliovirus receptor CD155, which was found to be upregulated in GBM and expressed on APCs. The neuropathogenicity of the virus is attributed to its internal ribosome entry site (IRES), which is substituted in the genetically modified version with that of human rhinovirus type 2 (HSV2) (105).

In preclinical glioma xenograft mouse models, PVSRIPO was demonstrated to be safe and resulted in significant tumor regression (105, 106). In a phase I trial (NCT01491893) with a dose-escalation phase and subsequent dose-expansion phase, 61 patients with recurrent GBM received intratumoral infusions of PVSRIPO by convection-enhanced delivery. Overall results were promising and confirmed the absence of neurovirulence. The only dose-limiting toxic effect that was reported was an intracranial hemorrhage that occurred immediately after removal of the catheter. In the dose-expansion phase, 19% of patients exhibited virus-related adverse events of grade 3 or higher. The achieved median overall survival of 12.5 months was not significantly different from that of the 11.3 months observed for historical treatments. However, the overall survival rate plateaued at 21% at 24 and 36 months, comparing favorably to the 14% at 24 months and 4% at 36 months observed in the historical control group. Preliminary immune evaluations from the trial suggested a reduction in immunosuppressive T_reg_ cell levels (107). These findings provided the rationale for further assessment of PVSRIPO against recurrent GBM in a phase II clinical trial (NCT02986178).

### 3.4 Retrovirus

#### 3.4.1 Retroviral replicating vector (Toca 511)

Retroviruses are enveloped RNA viruses. Toca 511 is a replicating gamma-retroviral vector encoding a yeast cytosine deaminase (CD) gene that catalyzes the conversion of the anti-fungal drug 5-flucytosine (5-FC) into the active chemotherapeutic agent 5-fluorouracil (5-FU), thereby eliciting a local anti-tumor response (108). Toca 511 has demonstrated strong oncolytic activity in preclinical models of glioma (108–110). Furthermore, high local concentration of 5-FU through Toca 511 have been shown to deplete immunosuppressive myeloid cells in the TME, resulting in the establishment of a T cell-mediated anti-tumor immune response (111, 112).

The safety and tolerability of Toca 511 and oral Toca FC were evaluated in three phase I clinical trials using three different routes of administration: intratumoral injection without resection (NCT01156584), injection into the walls of the resection cavity (NCT01470794), and intravenous administration followed by injection into the resection cavity (NCT01985256). In all trials, 4–6 weeks after administration of the final Toca 511 dose, patients were treated with Toca FC in repeated cycle every 4–8 weeks. Toca 511 was generally well tolerated in all patients across the trials, without dose-limiting toxicities (113).

The phase I dose-escalation study of intratumorally injected Toca 511 with orally administered Toca FC in patients with recurrent GBM and anaplastic astrocytoma who had undergone surgical resection (NCT01470794) showed promising results. A complete response was reported in 6 of 53 (11.3%) efficacy-evaluable patients and was maintained for a median 35.1 months, significantly longer than the duration observed for existing therapies, with a range of 2.79 to 11.6 months. A subset of 23 patients who matched the recommended phase III Toca 511 dose and patient eligibility appeared to derive the greatest benefit, with 5 patients (21.7%) achieving complete response and an overall clinical benefit rate of 43.5%. The median overall survival was 14.4 months (114). These results granted Toca 511 its initial fast-track designation by the FDA. Unfortunately, despite these encouraging results, the randomized phase II/III trial (NCT02414165) was suspended for failing to show a survival advantage compared with the standard of care for recurrent GBM patients (115).

### 3.5 Parvovirus

#### 3.5.1 Rat protoparvovirus H-1

Another completed clinical trial involved the evaluation of H-1PV, a single-stranded DNA virus whose natural host is the rat, as an oncolytic agent against GBM. H-1PV is non-pathogenic in humans, and cancer cells are susceptible to H-1PV infection due to their high levels of factors essential for cellular viral replication (116). Complete GBM tumor regression was observed in preclinical rat models (117). In a phase I/IIa clinical trial (NCT01301430), 18 patients with progressive primary or recurrent GBM received either an intratumoral or intravenous injection of PavOryx01 followed by tumor resection and a subsequent virus injection into the resection cavity. Treatment was generally well tolerated with no dose-limiting toxicity and one virus-related adverse effect. Results demonstrated the ability of H-1PV to cross the BBB to reach the tumor. Importantly, this OV resulted in a modulation of the TME indicated by strong CD8^+^ and CD4^+^ T-cell infiltration, decreased T_reg_ cells, and increased production of pro-inflammatory cytokines IFNγ and IL-2 in the treated patients. Median overall survival was 15.2 months, and progression-free survival was 15.9 months (118).

### 3.6 Measles virus

Measles virus (MV) is a negative single-stranded RNA virus that belongs to the family of Paramyxoviruses and has been shown to have oncolytic properties in a wide range of malignancies. Attenuated vaccine strains of MV are naturally oncolytic and have been engineered to enhance their tumor selectivity and allow their *in vivo* tracking. The MV Edmonton strain (MV-Edm) has been modified to express carcinoembryonic antigen (CEA) as a reporter gene for the *in vivo* monitoring of viral activity (119). Intratumoral treatment of glioma mouse models with MV-CEA resulted in significant tumor regression (120). A recently completed phase I clinical trial (NCT00390299) with results yet to be published investigated MV-CEA in 23 patients with recurrent GBM. In group A, MV-CEA was directly administered into the resection cavity, while in group B, MV-CEA intratumoral catheter administration was followed by resection and virus injection directly to the tumor bed. The maximum tolerable dose was 10^7^ pfu tissue culture infectious dose 50 (TCID_50_). For the primary outcome, 6/9 (67%) patients in group A experienced grade 3 or higher adverse effects versus 5/13 (39%) patients in group B. The median overall survival was reported to be 6 years.

### 3.7 Reovirus

REOLYSIN is an unmodified wildtype serotype-3 reovirus, a double-stranded RNA virus that is non-pathogenic in humans. REOLYSIN has been reported to be selective for tumor cells with activated Ras signaling (121). Reovirus tested in preclinical models showed direct tumor lysis in addition to enhanced T-cell infiltration and secretion of type I IFN (122). In a phase I dose-escalation trial, 12 patients with recurrent malignant glioma were intratumorally injected with REOLYSIN. One patient had stable disease and the median overall survival was 21 weeks. The treatment was well tolerated, and the maximum-tolerated dose was not reached (123). Similar results were observed for a phase I trial assessing intratumoral infusion of REOLYSIN *via* convection-enhanced delivery (CED) over 72 hours in 15 patients (NCT00528684). Stable disease was observed in three patients, and one patient showed partial response. This was the first trial to use CED for viral administration (124). Interestingly, a phase Ib clinical trial testing the intravenous administration of reovirus showed that tumors from reovirus-treated patients exhibited increased leukocyte infiltration, IFN expression, and PD-L1 expression (EudraCT 2011-005635-10) (122). REOLYSIN is currently being tested in a phase I trial in six pediatric patients with recurrent glioma. Patients will receive granulocyte-macrophage colony-stimulating factor (GM-CSF) on days 1 and 2, followed by intravenous infusions of REOLYSIN over 60 minutes on days 3–5. GM-CSF is expected to boost the anti-tumor immune response by inducing DC maturation and stimulating cross-presentation of tumor antigens (125).

### 3.8 Vaccinia virus

Vaccinia virus (VV) is an enveloped double-stranded DNA virus belonging to the *Poxviridae* family. TG6002 is a modified VV with a deletion in the thymidine kinase (TK) gene and the ribonucleotide reductase gene and is armed with the suicide gene (*FCU1*) to enhance tumor selectivity (126). TG6002 has exhibited oncolytic activity preclinically and is currently under clinical evaluation in combination with 5-FC in a phase I/II clinical trial with intravenously delivered virus in 78 GBM patients (NCT03294486) (126, 127).

In general, early-stage clinical trials of OVs have provided evidence of safety, tolerability, and favorable survival rates compared with conventional therapies. Results of ongoing and planned late-phase trials are eagerly anticipated. Clinical investigation of OVs have highlighted the profound tumor heterogeneity and low mutation load of GBM as limiting factors (128).

Oncolytic virotherapy is an emerging and continually evolving therapeutic platform in the treatment of many cancers. Despite the promising safety and efficacy data from ongoing clinical trials, identifying the optimal route, dosage and combination regimen with other immunotherapeutics is still under investigation. The immunosuppressive TME, the crossing of the blood brain barrier (BBB) and the tumour heterogeneity represent major barriers to the success of OV therapy in GBM. Further understanding of the interaction of the TME with OVs is critical to improve the spread of the virus and therapeutic efficiency. Moreover, performing molecular studies on tumour tissues before and after treatment will likely yield information necessary for identifying potential biomarkers correlated to sensitivity or resistance to OV therapy. A comprehensive review of potential biomarkers for OVs in GBM was recently published by Stabrakaki et al. (129). The recent approval of G47 from Japan’s MHLW for the treatment of GBM represents a critical milestone and pending results from ongoing clinical trials will certainly play a critical role in the shape of the landscape of treatment of GBM.

## 4 Non-replicating viral vectors for the treatment of glioblastoma

In gene therapy, vectors can be used as vehicles to deliver genetic material of therapeutic use into tumor cells to manipulate tumor genetics and produce a specific anti-tumor response. Administration of a vector for gene delivery in GBM is achievable, as the tumor can be accessed *via* neurosurgical means and advanced imaging models. The vectors could be injected systemically or permeated to adjacent parenchyma of GBM following a de-bulking surgery to selectively destroy GBM cells (130, 131).

Ideally, choosing a suitable vector for gene therapy is based on the nature of the target cells, the size of the genetic material that can be incorporated into the vector, and the ability of the vector to maintain long-time gene expression. Gene therapy vectors are broadly classified as viral or non-viral vectors (132). Viral vectors are valuable tools for gene therapy as they can be easily modified and possess the inherent property of horizontal gene transfer (133). They are most commonly used in cancer gene therapy due to their transfection efficiency and their vigorous cytotoxic effect on tumor cells (134). Progressive developments in vector engineering, delivery, and safety in the treatment of GBM have positioned viral vector-based therapy ahead of various other therapies.

In this section, we focus on the different types of viruses that have been developed for use as vectors for gene therapy in GBM, including Ad, adeno-associated virus (AAV), retrovirus, baculovirus, and lentivirus. We highlight the versatility of these viral vectors for GBM, describing some of their advantages and disadvantages. Replicating vectors release viral particles into the bloodstream that may cause an unavoidable immune response, and partial deletion of the viral genome to prevent replication allows for successful delivery of the therapeutic genes.

### 4.1 Adenovirus-based vectors

There are at least 57 serotypes of human Ad (Ad1–Ad57) in seven species (A–G) (135). Gene therapy applications using Ad have typically used Ad vectors originating from serotypes 2 and 5, classified under Ad type C (136). The genome of human Ad has five early-transcription units (E1A, E1B, E2, E3, and E4), four intermediate-transcription units, and one late-transcription unit (135). The genome of Ad does not integrate into the host genome; it remains episomal as an additional DNA element while expressing viral genes. Viral entry is CAR-dependent. The E1A gene product interacts with E2F-Rb or E2F-DP1 transcription complexes during the adenoviral replication cycle, forcing the infected cell into the S phase. This allows the virus to replicate its own genome using the cellular DNA replication machinery of the host cell (137).

To lower the toxicity of adenoviral infection, first-generation Ads were constructed by deleting the E1 and/or E3 region of the virus genome to eliminate the expression of viral genes within infected cells, and the therapeutic transgene was inserted into the E1 region. However, first-generation Ads contained residual viral proteins that caused a substantial immune response, impairing therapeutic transgene expression. Moreover, the insertion capacity for therapeutic transgenes was restricted to ~8 kbp (130). In addition, as these vectors do not have the ability to replicate, they become diluted after a few cycles of cancer cell proliferation; therefore, their expression drops rapidly. To target GBM using Ad vectors, deletion of critical viral genes that are supplied by tumor cells in trans is required. Furthermore, it is important to use tumor-specific promoters and modification of the viral capsid to permit selective entry of the vector into GBM cells (131).

High-capacity helper-dependent adenoviral vectors (HC-Ads) are the latest generation of Ads. They have been engineered to omit all endogenous viral-coding regions from the vector genome. These deletions reduce the immune response generated by HC-Ads compared with the first-generation vectors. In addition, they enable larger inserts with a maximum cloning capacity of ~35 kbp. Outstandingly, HC-Ads can elicit long-term transgene expression, even in the presence of an anti-Ad systemic immune response that has previously been shown to suppress the expression of transgenes from first-generation Ads (138). This adaptability enables the utilization of non-replicating Ads with fully engineered genomes for gene therapy applications in human patients with GBM.

Several preclinical studies have validated the safety and effectiveness of the administration of Ad into the brain of rodents and non-human primates. Moreover, Ad vectors are powerful vectors for the treatment of GBM due to their ability to attain high transgene expression levels. Clinical trials, including massive, multicenter, phase III clinical trials, have demonstrated that these vectors are a safe therapeutic approach (130).

#### 4.1.1 Adenoviral vector-based suicide gene therapy

Suicide gene therapy is a unique application of viral vectors that utilizes a form of gene-mediated cytotoxic immunotherapy (GMCI). Suicide genes encode conditionally cytotoxic enzymes that activate non-toxic compounds in a prodrug, incorporated by transduced cells, into cytotoxic molecules. These cytotoxic compounds cause damage and lysis of transgene-expressing cells and can freely diffuse into neighboring cells or migrate through cell-to-cell contact, amplifying their cytotoxic effect (so-called “bystander effect”) (139).

TK is the most exploited suicide gene in the treatment of GBM (140). Researchers have developed an adenoviral vector encoding the herpes simplex thymidine kinase (HSV-TK) gene, which has shown a promising effect in GBM in preclinical studies and clinical trials I/II. HSV-TK produces immunogenic proteins and interacts with antiviral drugs administered systemically (e.g., valacyclovir) to produce nucleotide analogs that interfere with tumor proliferation. Heterologous expression of HSV-TK phosphorylates the prodrug ganciclovir (GCV) to GCV-monophosphate, which is then converted to toxic GCV-triphosphate by tumor cell kinases and becomes 2-deoxyguanosine triphosphate. Incorporation of GCV-triphosphate into duplicating DNA leads to DNA chain termination through DNA polymerase inhibition (139).

A phase II clinical trial (NCT00870181) assessed the anti-tumor safety and efficacy of intraarterial cerebral infusion of replication-deficient Ad mutant thymidine kinase (Ad-TK) combined with systemic intravenous administration of GCV in recurrent high-grade glioma (rHGG) patients. The study demonstrated a significant improvement in the survival rate of the Ad-TK treated arm, with comparable efficacy and safety to other treatments for rHGG. Therefore, Ad-TK gene therapy is a valuable therapeutic approach for rHGG (141).

The cytotoxic effect of TK with GCV sensitizes GBM cells to radiotherapy and chemotherapeutic agents. Ad-TK administered pre-radiotherapy treatment effectivity improved radiotherapy in intracranial human GBM xenografts and decreased neurological side effects in mice, suggesting a synergistic effect between TK and radiotherapy (142).

Given the multiple preclinical and clinical trials showing TK synergism with cytotoxic agents and immune stimulants, it seems worthwhile to pursue its application further. Ongoing prospective phase I–II studies that began in 2018 aim to assess the effectiveness and safety of HSV-TK gene therapy with valacyclovir in combination with radiotherapy and standard-of-care chemotherapy for recurrent (NCT03596086) or newly diagnosed (NCT03603405) GBM or anaplastic astrocytoma patients. Moreover, combined delivery of genes encoding cytotoxic HSV-TK and human soluble fms-like tyrosine kinase ligand 3 (Flt3L) revealed persistence of anti-GBM immunological memory induced by this combination (143). Furthermore, to concentrate the toxin effect within the TME, the toxins were fused with ligands that bound to receptors overexpressed on the GBM cell surface, such as IL-13 (144).

Further development of the TK mutants SR39 and SR26 that exhibit high affinity for the prodrugs GCV and acyclovir, respectively, permitted the lowering of systemic concentrations of the prodrugs with suicide gene therapy, thereby minimizing toxicity (145). In addition, a novel tomato plant-derived TK (toTK), which exhibited high affinity and specificity for the nucleoside analogue azidothymidine (AZT), showed a robust cytotoxic effect in human GBM cells *in vitro*. AZT easily penetrated the BBB, phosphorylating AZT to AZT-monophosphate (146).

#### 4.1.2 Adenoviruses encoding an immunomodulatory molecule

The CNS is relatively separated from systemic immune responses. Therefore, it is challenging to trigger the immune system to induce a locoregional anti-tumor response against gliomas (147). Simultaneously, glioma cells are capable of suppressing and efficiently escaping cellular immune responses (148). To aid in the development of efficient immunotherapy for glioma, viruses have been engineered to express cytokines that activate immune cells and attract them to the tumor.

Human interleukin-12 (hIL-12) is a cytokine that can enhance anti-tumor immunity. IL-12 increases the tumor cell-destroying capabilities of the immune system, possibly *via* the interplay between the innate and adaptive immune responses. Unfortunately, the systemic application of IL-12 can cause toxic inflammatory responses and lead to multiorgan failure. To avoid this toxicity, a transcriptional switch can be used to control the dosing of hIL-12, such as the IL-12 oral activator Ad-RTS-hIL-12 (149). Ad-RTS-hIL12 is a recombinant replication-deficient serotype 5 Ad-encoding human pro-inflammatory interleukin 12 gene under the control of RheoSwitch Therapeutic System (RTS) promoter. Following a single vector injection, this engineered veledimex (VDX)-inducible promoter allows for long-term, uniform release of IL-12 in the tumor region. IL-12 stimulates the immune system, resulting in immune-mediated inhibition of cancer cell proliferation and tumor cell lysis (150). A multicenter phase I dose-escalation clinical trial (NCT02026271) evaluated the safety, tolerability, and biological effects of Ad-RTS-hIL-12 in 31 patients with recurrent high-grade glioma who underwent resection. A single injection of Ad-RTS-hIL-12 into the resection cavity walls with oral VDX administered preoperatively allowed the production of human IL-12. Ad-RTS-hIL-12 crossed the BBB, leading to an influx of activated immune cells into the TME. Increase VDX, IL-12, and IFNγ levels were observed in the peripheral blood in a dose-dependent manner, with ~40% VDX tumor penetration. Furthermore, VDX dosing controlled the frequency and intensity of adverse effects, including the cytokine release syndrome, with rapid reversal upon discontinued administration of the drug. VDX (20 mg) had a 12.7-month median overall survival in patients with rHGG and superior drug compliance compared with historical controls. Ad-RTS-hIL-12 increased tumor-infiltrating lymphocyte-mediated production of IFNγ, which supported an immunological anti-tumor effect. This phase I trial demonstrated acceptable tolerance of a regulated hIL-12 with encouraging preliminary results (149). A follow-up clinical study (NCT03330197) in the pediatric population aimed to assess the safety and tolerability of the single-tumor injection of Ad-RTS-hIL-12 combined with oral VDX. Another study (NCT03636477) added nivolumab to Ad-RTS-hIL-12 administered with VDX to aid the immune system in the detection and attack of cancer cells.

The hematopoietic growth factor Flt3L is a ligand for the Flt3 tyrosine kinase receptor, which is expressed on the surface of DCs. The absence of DCs from the brain parenchyma prevents the brain from mounting an immune response against gliomas. A recombinant replication-deficient serotype 5 Ad with CMV promoter-driven expression of human fms-like tyrosine kinase 3 ligand (Ad-hCMV-Flt3L) provides an immunomodulatory effect. Flt3L is a cytokine that stimulates the proliferation and migration of DCs to the tumor site. The vector is typically used in combination with other conventional therapies to increase the immune response to GBM by releasing Flt3L from damaged cells (151).

Another example of an Ad encoding an immunomodulatory molecule is BG00001. It is a replication-defective, recombinant Ad expressing the IFNβ (rAd-hIFNβ) gene. In a clinical trial (NCT00031083), rAd-hIFNβ was intratumorally injected in patients with recurrent grade III and IV gliomas. The study aimed to assess the efficacy and safety of injecting BG00001 into brain tumors.

#### 4.1.3 Adeno-based cancer gene therapy

SCH-58500 consists of intratumoral injection of manipulated Ad carrying the p53 gene into tumor cells in the brain, enhancing their immunogenicity. A phase I trial (NCT00004080) aimed to assess the effectiveness of SCH-58500 in the treatment of patients who had recurrent or progressive GBM, anaplastic astrocytoma, or anaplastic mixed malignant glioma that could be removed during surgery. The study included evaluation of the effectiveness of SCH-58500 on the TME at the molecular level. Furthermore, this clinical trial aimed to determine the maximum-tolerated dose of SCH-58500. In a dose-escalation study, patients received an initial intratumoral stereotactic injection of SCH-58500 followed by tumor resection. Then, the patients received a series of 1-minute injections of SCH-58500 into the resected tumor cavity wall. Patients were monitored closely for 12 weeks, then every 2 weeks for 8 weeks, every month for 8 weeks, and then every 2 months until death. A total of 30 patients were recruited for this study.

#### 4.1.4 Adeno-based cancer gene targeting using RNA interference

Selective degradation of mRNA by RNA interference (RNAi) is a promising approach. RNAi is a sequence-specific, posttranscriptional gene-silencing machinery whereby small interfering dsRNA lead to the degradation of mRNA homologous in sequence to that of the expression vectors, using U6 or H1 promoters. Gene targeting by siRNA is effective and specific. The application of siRNA technology to gene therapy is still a novel technique in the field; there is a need to increase the transduction efficiency of siRNA into target cells (152).

Uchida et al. developed an Ad vector with a tandem-type siRNA expression unit targeting survivin (Adv-siSurv). Survivin is a protein that has been shown to promote cancer progression and drug resistance. It is a promising selective target as it is commonly overexpressed in malignancies; however, it is undetectable in terminally-differentiated adult tissues. This Ad vector harbors a tandem-type siRNA expression unit; sense and antisense strands composing the siRNA duplex were independently transcribed by two human U6 promoters. Infecting cancer cells *in vivo* and *in vitro* with Adv-siSurv led to effective downregulation of survivin, remarkably reduced tumor growth, and induced apoptosis in many cell lines, including HeLa, U251, and MCF-7. Moreover, intratumoral injection of Adv-siSurv significantly suppressed tumor growth in a xenograft model using U251 glioma cells. Therefore, this novel modality may be a promising tool in cancer therapy (152).

In another Ad vector, Lakka et al. demonstrated that matrix metalloproteinase 9 (MMP-9) contributed to maintaining the invasiveness of glioblastoma. The study investigated an adenoviral vector carrying an antisense cDNA sequence to the 5′ end of the human MMP-9 gene (Ad-MMP-9AS) to downregulate the activity of MMP-9, limiting tumor metastasis in both *in vitro* and *in vivo* models (153).

#### 4.1.5 Combining treatments

To achieve maximum efficacy, several non-replicating Ad vectors have been combined. An example of Ad vector combination therapy is Flt3L and HSV1-TK intraparenchymal injection (with GCV). This combination therapy was shown to remodel the CNS immune microenvironment to induce an anti-tumor response. A preclinical study demonstrated persistence of the anti-GBM immunological memory induced by this combination. A multicenter, open-label, dose-escalation phase I safety study (NCT01811992) delivered Ad-hCMV-TK and Ad-hCMV-Flt3L first-generation adenoviral vectors to the peritumoral region after tumor resection. The principle behind this therapy is that the TK of the Ad-hCMV-TK converts GCV into phospho-GCV, which becomes cytotoxic to the transduced brain cells. Then, the exposed tumor antigen released from dying glioma cells is taken up by DCs and recruited to the peritumoral brain TME by Ad-hCMV-Flt3L expressing the cytokine Flt3L. This combined therapy is also expected to mediate a specific anti-tumor immune response against the remaining malignant glioma cells (151).

Another example of combination therapy using Ad vectors includes the co-delivery of reduced expression in immortalized cells/Dickkopf-3 (REIC/Dkk-3) and cyclic arginine-glycine-aspartate (cRGD). cRGD is an antagonist of integrins, which is overexpressed in cancer cells, and plays a significant role in angiogenesis and invasion in GBM. Tetsuo Ok et al. demonstrated that utilizing an Ad vector expressing REIC/Dkk-3 enhanced apoptotic cell death in human and murine GBM cell lines (148). Using the adenoviral delivery system to overexpress both REIC/Dkk-3 and cRGD led to a significant reduction in the tumor proliferation rate (154, 155).

### 4.2 Adeno-associated virus-based vectors

Initially, adeno-associated virus (AVV) human parvoviruses were identified as contaminants in Ad preparations using human or simian Ad stocks. Later, they were identified as a preferred candidate for gene therapy due to their non-pathogenic characteristics (156). There are 12 distinct AVV serotypes (AAV1–12) in human and non-human primates; AAV serotype 2 is the most common in gene therapy. AVVs are replication incompetent and require a helper virus to replicate in mammalian cells (157). Development of AAV vectors for gene therapy has allowed for a broad spectrum of therapeutic applications.

Non-replicating AAV-IL-12 has been shown to cause local immune induction in experimental models of GBM, increasing IFNγ expression, microglial activation, and recruitment of T and NK lymphocytes, resulting in a substantial anti-tumor effect (158).

Zhang et al. focused on enhancing the selectivity of AAV2 vectors against U87 glioblastoma cells in suicide gene therapy by designing vectors with favorable conditions to conjugate fluorophores bio-orthogonally and cRGD peptides stoichiometrically in AAV. These modifications improved the selective tropism of AAV vectors toward integrin-expressing GBM tumor cells, increasing the efficacy of HSV-TK gene/GCV therapy 25-fold (159).

Intracranial administration of AAVs encoding *IFNβ* has been also shown to treat invasive human GBM8 tumors in xenograft and GL261 tumors in syngeneic mouse models. AAV with mouse IFNβ vector administration in the syngeneic mouse tumor model increased the median survival rate up to 56% compared with the control group (160).

dsAAV2 was developed to overcome the slow expression of single-stranded AAV. dsAAV-decorin is a dsAAV2 that delivers stable and high-level decorin expression to cancer cells. Decorin is a small leucine-rich proteoglycan that has anti-cancer activity; dsAAV-decorin has been reported to significantly inhibit malignant U87MG glioma growth *in vivo*. Proteomics analysis suggested that dsAAV-decorin induced the differentiation of glioma cells by multiple biochemical mechanisms, rendering human glioma cells vulnerable to chemical or radiation therapies. Therefore, dsAAV-decorin is a potential candidate for gene therapy in malignant glioma patients (161).

### 4.3 Herpes simplex virus-based vectors

The epidermal growth factor receptor (EGFR) is often altered in glioblastoma, implying its critical function in glial tumorigenesis and progression. HSV-1-based amplicons were developed to include the RNA polymerase III-dependent H1 promoter to enable the expression of the double-stranded hairpin RNA against EGFR at two different locations (pHSVsiEGFR I and pHSVsiEGFR II). This posttranscriptional gene silencing by vector-mediated RNAi knocked down EGFR in a dose-dependent manner, inhibiting the growth of human glioblastoma (gli36-luc) cells both *in vitro* and *in vivo* (162). These observations suggest that HSV-1 amplicons can produce effective posttranscriptional gene silencing.

### 4.4 Lentivirus-based vectors

Lentiviral vectors are capable of transducing quiescent, non-dividing cells (163); therefore, they are promising candidates for the treatment of brain cancer. Lentiviral vectors have been engineered to contain a small hairpin RNA (shRNA) to silence sirtuin 1 (SirT1) expression in glioblastoma-derived cells. SirT1 promotes tumorigenesis and inhibits apoptosis; downregulating SirT1 *via* this shRNA-expressing vector has been shown to enhance tumor sensitivity toward radiotherapy-induced tumor death. This therapy increased the mean survival rate of nude mice transplanted with CD133^+^ GBM cells (164).

Similarly, glioblastoma stem cells were transduced with lentiviral vectors expressing human orphan nuclear receptor tailless (TLX) shRNA. TLX is critical for maintaining tumor growth and self-renewal. Upon downregulation of TLX, the growth and self-renewal properties of the glioblastoma stem cells were inhibited. Moreover, downregulating TLX induced the expression of methylcytosine dioxygenase 3 (TET3), a tumor-suppressor gene (165).

### 4.5 Retrovirus-based vectors

Various *in vivo* and *in vitro* studies have illustrated the ease of using retrovirus-mediated gene transduction to kill glioma cells. In 1991, the first clinical trial of gene therapy for GBM was conducted using retrovirus-producing cells. A retroviral vector encoding HSV-TK was administered at a specific cerebral stereotaxic position in combination with GCV, resulting in GBM tumor remission. However, this study showed that retroviral vectors had limited transfection efficiency (166).

### 4.6 Baculovirus-based vectors

Baculoviruses are widely present in nature and have been extensively researched for their biology and biosafety. Baculoviruses have unique characteristics; they contain a 130-kb viral genome. This large cloning capacity allows for the delivery of one large functional gene or multiple genes from a single vector. Furthermore, because baculoviruses do not express mammalian promoters, they can enter mammalian cells but cannot replicate in them. Recombinant baculoviruses were developed to express mammalian promoters, allowing for high transduction efficiency (167, 168).

A recombinant baculovirus viral gene delivery system was developed to minimize possible side effects caused by overexpression of a therapeutic gene in non-target cells. Astrocyte-specific baculovirus was one of the initial attempts at using baculovirus in cancer gene therapy to treat malignant glioma. As glial fibrillary acidic protein (GFAP) is expressed abundantly and almost exclusively in astrocytes of the CNS, the development of a recombinant baculovirus vector accommodating the engineered GFAP promoter was shown to drive astrocyte-specific expression in cultured cells. This approach reduced possible side effects from overexpression of a therapeutic gene in sensitive neurons, and it effectively suppressed tumor development in a rat xenograft model (169).

As mentioned previously, tissue-specific cellular promoters have proven be effective transcriptional targeting methods for limiting transgene expression in targeted tissues. GFAP^+^ nontumor glial cells have a relatively lower rate of proliferation than tumor cells, making them less susceptible to DNA synthesis inhibition by phosphorylated GCV. Nevertheless, there is a high demand to protect normal glial cells, which are abundant in both the CNS and the peripheral nervous system. To minimize the killing of non-target, normal astrocytes, Wu et al. used the baculovirus viral gene delivery system with microRNA (miRNA) posttranscriptional regulation in addition to the GFAP tissue-specific cellular promoter. Baculoviral vectors containing the HSV-TK suicide gene were constructed to be under the control of the engineered GFAP promoter that could restrict transgene expression to the glial cell lineage in the vectors. The vectors also carried repeated target sequences of three endogenous miRNAs that were found to be downregulated in glioblastoma cells as compared with astrocytes. This modification led to substantial enhancement of *in vivo* selectivity, allowing for successful eradication of human glioma xenografts with minor toxic effects on normal astrocytes. Therefore, miRNA integration into a transcriptional targeting vector reduces off-target transgene expression. This approach of transcriptional targeting is likely to improve the specificity of cancer suicide gene therapy (170).

### 4.7 Parvovirus-based vectors

Recombinant parvoviruses have been also used in an attempt to modulate the immune system of GBM. Enderlin et al. used parvoviruses to transduce IFNγ-inducible protein 10 (CXCL10) and TNF-alpha cytokines concurrently in a syngeneic mouse model of GBM. CXCL10 stimulates the recruitment of activated T and NK lymphocytes to the tumor and inhibits tumor angiogenesis. In contrast, TNF-alpha promotes DC maturation and leads to tumor necrosis. Transducing cells with both cytokines prior to implantation led to a synergistic effect of both vectors, causing regression of the tumors. There was a delay in tumor growth in naïve, pre-established tumors; however, no regression was observed (171).

Viral-vector gene therapy has shown strong therapeutic potential in GBM treatment in clinical trials but has not achieved FDA approval, yet. Issues with limited efficacy, viral delivery, inefficient tumor penetration, and some safety concerns regarding the full extent of their clinical impact on the long term require optimizations and further development. Nevertheless, the wealth of innovative solutions being explored across academia, biotech, pharma and manufacturing organizations assures that viral-vector gene therapies are very promising.

## 5 CAR-T-cell therapy for the treatment of glioblastoma

CAR is a synthetic antigen-specific receptor engineered on the surface of an immune cell. As their name implies, CARs are chimeric molecules. They are composed of three fragments: a targeting moiety, usually in the form of a single-chain variable fragment (scFv); a transmembrane domain; and an intracellular signaling domain. The interaction between a CAR molecule and its target can result in the activation of an antigen-specific immune response (172, 173).

The nature of the intracellular signaling endodomain determines the CAR “generation”. First-generation CARs contain a single signaling domain, which is typically CD3ζ. The inclusion of a single co-stimulatory domain upstream of this activating sequence resulted in the development of second-generation CARs. This co-stimulatory domain is most commonly derived from CD28 or 4-1BB (174–178), and the inclusion of two co-stimulatory sequences fused in tandem and inserted upstream of CD3ζ is referred to as a third-generation CAR (179).

Engineering CAR-T cells involves the isolation of T cells from peripheral blood mononuclear cells followed by *ex vivo* gene modification using viral transduction or electroporated transposon vectors. Then, engineered CAR-T cells are generally expanded *ex vivo* prior to infusion into patients (172).

Recently, CAR-T-cell therapy has been investigated preclinically and in early-phase clinical trials to evaluate the feasibility and safety profiles of this technology for glioblastoma. Despite reports of unprecedented clinical responses in patients with hematologic malignancies following the use of genetically-engineered CAR-T cells, the efficacy of CAR therapy for solid tumors, including GBM, remains challenging. As mentioned earlier, the brain is a critical organ; the selection of therapeutic candidates for CAR-T cells requires caution and evidence of negligible expression in normal brain tissues to avoid on-target off-tumor toxicity. Therefore, only a limited number of CAR candidates have been studied in humans with glioblastoma: interleukin-13 receptor subunit alpha 2 (IL-13Ra2), human epidermal growth factor receptor 2 (HER2), epidermal growth factor receptor variant III (EGFRvIII), disialoganglioside (GD2), erythropoietin-producing hepatocellular carcinoma A2 (EphA2), B7-H3, chlorotoxin (CLTX), natural-killer group 2D ligands (NKG2DLs), CD133, CD70 (180), chondroitin sulfate proteoglycan 4 (CSPG4) (181), carbonic anhydrase IX (CAIX) (182), ephrin type A receptor 2 (EphA2) (183), and integrin αvβ3 (184). Some of these CAR-T-cell targets are discussed below and they are summarized in **Supplementary Table 4**.

### 5.1 IL-13 Ra2 CAR-T cells

The IL-13 Ra2 receptor subunit is highly expressed in glioblastoma but not in healthy brain cells (185), making it an ideal target for CAR-T-cell therapy. A first-generation CAR specific for IL-13 Ra2, named IL-13 zetakine, was generated to evaluate its feasibility and safety in patients with glioblastoma (186). In clinical trials, nine patients with recurrent glioblastoma received repeat doses of autologous (3 patients) and allogeneic (6 patients) IL-13 zetakine CD8^+^ T-cell clones intratumorally (intracranial tumoral) (187–189). These early clinical trials showed that IL-13 Ra2 CAR could be successfully manufactured and delivered to glioblastoma patients thorough a reservoir/catheter system. The therapy was well tolerated in all nine patients, and no dose-limiting toxicity was observed. Furthermore, signs of transient anti-glioma activity were detected by an increase in necrotic tumor volume and a decrease in the expression of IL-13 Ra2. Nevertheless, as with all other first-generation CAR products, the persistence of the engineered T cells was limited. Therefore, an optimized second-generation CAR, containing 4-1BB co-stimulatory domain, was developed. In a pilot clinical study, a patient with recurrent multifocal glioblastoma was treated with the second-generation IL-13 Ra2 CAR-T cells over 220 days, first through the intracranial tumoral route, followed by infusions delivered intraventricularly. All infusions of CAR therapy were well tolerated, without any toxic effects of grade 3 or higher. Importantly, this CAR-T-cell product demonstrated increased persistence and anti-tumor activity that was correlated with the clinical outcomes. Moreover, the patient had regression of all intracranial and spinal tumors, which persisted for 7.5 months (190). This case report highlights the potential safety and therapeutic efficacy of CAR-T cells for the treatment of glioblastoma.

### 5.2 HER2 CAR-T cells

HER2 is a transmembrane TK receptor overexpressed in a myriad of cancers (191). While HER2 overexpression has been reported in nearly 80% of glioblastoma patients, the receptor is not observed in healthy brain tissue (192), making it an ideal CAR candidate for glioblastoma. In a dose-escalation clinical phase I study, 17 patients with progressive HER2^+^ glioblastoma received one or repeat doses of peripherally infused autologous second-generation HER2 CAR-T cells. Although infusions were well tolerated with no dose-limiting toxicity, two patients experienced grade 2 seizures or headaches. Of the 16 evaluable patients, 1 experienced partial response for over 9 months, 7 had stable disease for 8 weeks to 29 months (3 of them remained progression-free during the 24–29 months of follow-up), and 8 progressed after therapy. Although this trial showed initial evidence of safety and a transient clinical benefit of HER2 CAR-T cells, it reported that the CAR-T cells did not expand after infusion and persisted only at a low frequency, suggesting the need for improved CAR engineering (193). Later, a preclinical study demonstrated that replacing the co-stimulatory CD28 domain with 4-1BB could improve the anti-tumor activity of HER2 CAR-T cells (194). This encouraging finding is currently under clinical evaluation in recurrent or refractory patients with grade II–IV glioma (NCT03389230).

### 5.3 EGFRvIII CAR-T cells

EGFRvIII is a splice variant of the TK receptor EGFR. EGFRvIII is expressed in nearly 20% of glioblastoma cases and is associated with disease development and progression (195). O’Rourke et al. conducted a clinical trial that involved treating 10 patients diagnosed with recurrent glioblastoma with a single intravenous dose of EGFRvIII CAR-T cells (196). Given that no major adverse events were reported, the study provided initial feasibility and safety of the treatment. However, none of the participants had tumor regression, with only one patient had stable disease for more than 18 months. Later, in a dose-escalation phase I trial, third-generation CAR-T cells against EGFRvIII, combined with intravenous IL-2, were used in the treatment of 18 patients diagnosed with recurrent glioblastoma expressing EGFRvIII. Of the 18 treated patients, no objective responses were reported, and the median progression-free survival was only 1.3 months. Furthermore, two patients developed severe hypoxia, including one treatment-related mortality after cell infusion at the highest dose level (197). This study highlights the challenges associated with treating GBM using targeted cellular therapy and highlights the importance of finding more specific CAR targets.

### 5.4 GD2 CAR-T cells

GD2 is a glycosphingolipid expressed by various types of malignant cells, including glioblastoma, and it is involved in tumor growth and invasion (198). Using GD2 CAR-T cells against patient-derived substitution mutation of lysine for methionine at position 27 in a histone H3 (H3K27M) glioma cell line that highly expressed GD2 resulted in potent glioma cell killing *in vitro*. This observation was supported by five independent patient-derived H3K27M diffuse-mudline glioma orthotopic xenograft mouse models that demonstrated a robust anti-tumor effect mediated by GD2 CAR-T cells (199). Currently, there are several phases I clinical trials investigating the safety and efficacy of GD2-specific CAR-T cell therapy in patients with different types of glioma (NCT04196413, NCT03423992, NCT04099797).

### 5.5 B7-H3 CAR-T cells

B7-H3 (CD276) is a checkpoint molecule that belongs to the B7 superfamily. It is upregulated in high-grade gliomas, and it has been shown to facilitate tumor cell migration and invasion (200). Employing B7-H3-specific CAR-T cells against pediatric solid tumors and brain tumors in preclinical mouse models resulted in a significant survival advantage and potent anti-tumor effect (201–203). Moreover, B7-H3 CAR-T cells injected intratumorally in glioblastoma-bearing mice resulted in sustained tumor regression for more than 120 days (203). Several clinical trials are currently ongoing to evaluate B7-H3 CAR-T-cell therapy for patients with malignant gliomas (NCT04077866, NCT04185038, NCT04385173).

### 5.6 CLTX CAR-T cells

CLTX is a small amino acid scorpion-derived peptide. Although the precise cell-surface receptor for CLTX remains to be identified, it possesses targeting properties toward cancer cells, including glioblastoma cells. Driven by this observation, it was hypothesized that CLTX could be employed for tumor-specific delivery of CAR-T cells. Interestingly, as CLTX is not expressed by cancer cells but rather a targeting molecule, this approach aims to overcome GBM tumor heterogeneity (204). Early clinical data demonstrated robust anti-glioblastoma activity in orthotopic xenograft glioblastoma models. Using different delivery routes, intracranial and intracerebroventricular CLTX CAR-T-cell administration resulted in tumor regression that persisted for over 170 days, whereas mice treated intravenously did not achieve similar tumor elimination (205). A phase I clinical trial using CLTX-CAR is currently recruiting patients with recurrent glioblastoma (NCT04214392).

### 5.7 NKG2DLs CAR-T cells

NKG2DLs are induced self-proteins that present at low levels in normal cells but are upregulated in most cancers, including glioblastoma (206). The use of NKG2D-based CAR-T cells *in vitro* showed efficient destruction of glioblastoma cells and glioblastoma stem cells accompanied by high levels of cytokine, granzyme B, and perforin secretion (207, 208). Delivering NKG2D CAR-T-cell therapy systemically in a fully immunocompetent orthotopic glioblastoma mouse model demonstrated homing of CAR-T cells to the tumor site in the brain, prolonged survival, cured fraction of glioma-bearing mice, and no reported significant adverse events (208). Similarly, Yang and colleagues demonstrated tumor eradication in glioblastoma-bearing NOD.CB17-PrkdcscidIl2rgtm1/Bcgen (B-NDG) mice after injecting NKG2D-based CAR-T cells intravenously (207). Because both studies supported the potential therapeutic gain of NKG2D CAR-T cells in glioblastoma, this therapy is currently under clinical evaluation in patients with relapsed or refractory GBM (NCT04717999).

### 5.8 CD133 CAR-T cells

CD133 is a transmembrane protein that serves as a cancer stem cell marker and is commonly upregulated in glioblastoma (209). Zhu et al. reported that treating patient-derived glioblastoma stem cells both *in vitro* and in orthotopic glioma murine models with CD133 CAR-T cells could result in tumor eradication (210). This observation was further supported by engineering a PD-1-deficient CD133-specific CAR-T cells that induced sustained levels of cytokine secretion and persistent proliferation with enhanced anti-tumor efficacy in the U251 xenograft GBM model (211). A pilot clinical study to assess the safety and efficacy of CD133 CAR-T cells is currently in the recruitment stage for patients with recurrent malignant gliomas (NCT03423992).

### 5.9 Multi-antigen-targeted CAR-T cells

Despite the growing number of clinical trials testing CAR-T therapy against different GBM antigens, this treatment strategy has failed to achieve complete remission in a clinical setting. Therefore, it was suggested that simultaneously targeting two or more tumor-associated antigens expressed on the surface of glioblastoma cells could increase CAR specificity, thus improving patient outcomes. Supporting evidence came from the observation that T cells co-expressing CARs against HER2 and IL-13 Ra2 exhibited enhanced anti-tumor efficacy in an orthotopic xenogeneic mouse model (212, 213). In addition to combinational targeting of these two receptors, CAR-T cells targeting three molecules simultaneously (HER2, IL-13 Ra2, and EphA2) were engineered to overcome interpatient antigenic variability and to improve clinical outcomes (214).

To further improve CAR-T-cell activity, CAR-T cells were engineered to express cytokines, chemokines, or their receptors to stimulate T-cell proliferation and persistence. Different research groups have modified CAR designs to secrete IL-15, IL-7R, IL-12, or IL-18, all of which have been shown to enhance CAR anti-tumor activity and persistence in glioblastoma (215–217). Although these modifications have improved therapy outcomes, they pose the risk of uncontrollable T-cell proliferation, which could lead to unintended toxicity. As such, CAR-T cells are being investigated to express an inducible suicide gene as a safety switch. For example, Yi et al. employed this approach with EphA2 CAR-T cells *via* the inclusion of a CD20 tag in the CAR vector to treat glioblastoma. Upon adding rituximab, a monoclonal antibody that targets CD20, the activated CAR-T cells were abolished within 24 hours (183).

The number of ongoing CAR studies both preclinically and clinically to tackle glioblastoma highlights the challenges facing the technology, mainly due to the mechanisms of immune escape such as antigen loss and immunosuppressive tumor microenvironment. These mechanisms have compelled scientists to target different antigens and frequently dual and triple targets at once as outlined above. The investigators have also developed multiple CAR designs to enhance their proliferation, trafficking, infiltration and function. This all shows the complexity of treating glioblastoma and emphasizes the importance of developing further strategies/combining different therapies such as immune checkpoint inhibitors to overcome the obstacles in order to treat this refractory disease.

## 6 Conclusion

Over the past decades, tremendous progress in various innovative immunotherapy strategies was revealed by a significant number of GBM preclinical studies and clinical trials. In this review, we discussed in detail the current clinical progress of the major types of GBM immunotherapies, including immune checkpoint inhibitors, oncolytic virotherapy, non-replicating viral vectors, and CAR-T-cell therapy. Other immunotherapies that have also been extensively investigated for the treatment of GBM include cancer vaccines and gut microbiota (7, 8). This disease remains one of the most difficult to treat, and to date no immunotherapy has been granted regulatory approval, except for the MSI-H/dMMR/TMB-H rare GBM cases. Multiple factors are potentially responsible for this problem, including the complex biology of GBM, the lack of reliable biomarkers and assessment tools for immunotherapy clinical trials (218), and the risk of CNS toxicity as a result of vigorous immune response (219, 220). While ongoing clinical studies are directed toward combination therapy, pre-clinical studies currently employ advanced technologies to unravel the complex and dynamic immunosuppressive GBM TME (221). We have a lot of work ahead of us, but the future of GBM immunotherapy seems promising.

## Author contributions

AM and AA, contributed to study concept and design. RA, SA, MD, MA, MT, and SAA collected and sorted out literatures. AM, RA, and AA drew pictures. SA, MD, MA, MT, and SAA wrote the first draft. AH and RA Validation and Editing. AM and AA Reviewing and Supervision. All authors contributed to the article and approved the submitted version.

## Funding

The authors extend their appreciation to the Deputyship for Research& Innovation, Ministry of Education in Saudi Arabia for funding this research work through the project number (918).

## Acknowledgments

The authors would like to extend their appreciation to Taibah University for its supervision support. **Figure 3** was created with BioRender (BioRender.com).

## Conflict of interest

The authors declare that the research was conducted in the absence of any commercial or financial relationships that could be construed as a potential conflict of interest.

## Publisher’s note

All claims expressed in this article are solely those of the authors and do not necessarily represent those of their affiliated organizations, or those of the publisher, the editors and the reviewers. Any product that may be evaluated in this article, or claim that may be made by its manufacturer, is not guaranteed or endorsed by the publisher.

## Supplementary material

Supplementary Table 1 Clinical trials of checkpoint inhibitors for the treatment of GBM.

Supplementary Table 2 Completed clinical trials of oncolytic virotherapy for the treatment of GBM.

Supplementary Table 3 Currently active and/or recruiting clinical trials of oncolytic virotherapy for the treatment of GBM.

Supplementary Table 4 Currently active and/or recruiting clinical trials of CAR-T cell therapy for the treatment of GBM.
