# Supplementary material for: Advances in immunotherapy for glioblastoma multiforme
Source: Front Immunol. 2022 Oct 12;13:944452. doi: 10.3389/fimmu.2022.944452 (PMC9597698; doi:10.3389/fimmu.2022.944452)
Supplement: Supplementary Table 1 — Clinical trials of checkpoint inhibitors for the treatment of GBM. [file DataSheet_1.pdf]

Supplementary Table 1. Clinical trials of checkpoint inhibitors for the treatment of GBM.

	Trial Number	Phase	Immune Checkpoint	Interventions	Status
1	NCT02667587	III	PD-1	Drug: Nivolumab<br>Drug: Temozolomide<br>Radiation: Radiotherapy<br>Other: Nivolumab Placebo	Active, not recruiting
2	NCT02617589	III	PD-1	Drug: Nivolumab<br>Drug: Temozolomide<br>Radiation: Radiotherapy	Active, not recruiting
3	NCT02017717	III	PD-1<br>CTLA-4	Biological: Nivolumab<br>Biological: Bevacizumab<br>Biological: Ipilimumab	Active, not recruiting
4	NCT04396860	II<br>III	CTLA-4<br>PD-1	Biological: Ipilimumab<br>Biological: Nivolumab<br>Radiation: Radiation Therapy<br>Drug: Temozolomide	Recruiting
5	NCT04479241	II	PD-1	Biological: PVSRIPO,<br>Biological: Pembrolizumab	Recruiting
6	NCT03890952	II	PD-1	Drug: Nivolumab,<br>Drug: Bevacizumab	Recruiting
7	NCT03047473	II	PD-L1	Biological: Avelumab	Active, not recruiting
8	NCT02798406	II	PD-1	Biological: DNX-2401,<br>Biological: Pembrolizumab	Active, not recruiting
9	NCT03014804	II	PD-1	Biological: autologous dendritic cells pulsed with tumor lysate antigen Vaccine,<br>Biological: Nivolumab,	Withdrawn
10	NCT04049669	II	IDO	Drug: Indoximod,<br>Radiation: Partial Radiation,<br>Radiation: Full-dose Radiation,<br>Drug: Temozolomide,<br>Drug: Cyclophosphamide,<br>Drug: Etoposide,<br>Drug: Lomustine	Recruiting
11	NCT02794883	II	PD-L1<br>CTLA-4	Biological: Durvalumab<br>Procedure: Surgical Procedure<br>Biological: Tremelimumab	Active, not recruiting
12	NCT04145115	II	CTLA-4<br>PD-1	Biological: Ipilimumab<br>Biological: Nivolumab	Recruiting
13	NCT04583020	II	PD-1	Drug: Camrelizumab<br>Radiation: radiation<br>Drug: Temozolomide	Recruiting
14	NCT04225039	II	Anti-GITR<br>Anti-PD1	Drug: INCMGA00012<br>Drug: INCAGN01876<br>Drug: SRS<br>Procedure: Brain surgery	Recruiting
15	NCT02550249	II	PD-1	Drug: Nivolumab	Completed
16	NCT03899857	II	PD-1	Drug: Pembrolizumab	Recruiting
17	NCT03291314	II	PD-L1	Drug: Axitinib<br>Drug: Avelumab	Completed
18	NCT02866747	I<br>II	PD-L1	Radiation: Hypofractionated stereotactic radiation therapy<br>Drug: Durvalumab	Recruiting

19	NCT04077866	I<br>II	B7-H3	Drug: Temozolomide,<br>Biological: B7-H3 CAR-T	Recruiting
20	NCT03277638	I<br>II	PD-1	Drug: Pembrolizumab at 7 days prior,<br>Drug: Pembrolizumab at 14 days post,<br>Drug: Pembrolizumab at 35 days post,<br>Procedure: Laser Interstitial Thermotherapy	Recruiting
21	NCT03750071	I<br>II	PD-L1	Biological: VXM01,<br>Biological: Avelumab	Recruiting
22	NCT03491683	I<br>II	PD-1	Biological: INO-5401,<br>Biological: INO-9012,<br>Biological: Cemiplimab,<br>Radiation: Radiation Therapy,<br>Drug: Temozolomide	Active, not recruiting
23	NCT04116658	I<br>II	PD-1	Biological: Multiple dose of EO2401 in combination with nivolumab	Recruiting
24	NCT02052648	I<br>II	IDO	Drug: Indoximod,<br>Drug: Temozolomide,<br>Drug: Bevacizumab,<br>Radiation: Stereotactic Radiation	Completed
25	NCT03347097	Early I	PD-1	Drug: TIL<br>Drug: PD1-TIL	Recruiting
26	NCT03707457	I	PD-1<br>GITR<br>IDO1<br>CTLA-4	Drug: Nivolumab,<br>Drug: Anti-GITR Monoclonal Antibody MK-4166,<br>Drug: IDO1 inhibitor INCB024360,<br>Drug: Ipilimumab	Terminated
27	NCT03726515	I	PD-1	Biological: CART-EGFRvIII T cells<br>Biological: Pembrolizumab	Active, not recruiting
28	NCT04003649	I	CTLA-4<br>PD-1	Biological: IL13Ralpha2-specific Hinge-optimized 4-1BB-co-stimulatory CAR/Truncated CD19-expressing Autologous TN/MEM Cells<br>Biological: Ipilimumab<br>Biological: Nivolumab	Recruiting
29	NCT03341806	I	PD-L1	Drug: Avelumab<br>Combination Product: MRI-guided LITT therapy	Recruiting
30	NCT04201873	I	PD-1	Biological: Dendritic Cell Tumor Cell Lysate Vaccine<br>Biological: Pembrolizumab<br>Drug: Poly ICLC	Recruiting
31	NCT02311920	I	CTLA-4<br>PD-1	Biological: Ipilimumab<br>Biological: Nivolumab<br>Drug: Temozolomide	Active, not recruiting
32	NCT04606316	I	PD-1<br>CTLA-4	Drug: Nivolumab-Placebo<br>Drug: Nivolumab<br>Drug: Ipilimumab-Placebo<br>Drug: Ipilimumab<br>Procedure: Surgery	Recruiting
33	NCT03233152	I	CTLA-4<br>PD-1	Drug: Ipilimumab (YervoyTM, 50 mg/10 mL solution)<br>Drug: Nivolumab (OpdivoTM, 40 mg/4mL solution)	Recruiting
34	NCT03961971	I	Tim-3<br>PD-1	Drug: MBG453	Recruiting

35	NCT02658981	I	LAG-3<br>PD-1<br>TNFRSF9/(CD137)	Biological: Anti-LAG-3 Monoclonal Antibody BMS 986016,<br>Biological: Anti-PD-1,<br>Biological: Anti-CD137	Active, not recruiting
36	NCT03493932	I	PD-1<br>LAG-3	Drug: Nivolumab,<br>Drug: BMS-986016	Recruiting
37	NCT04656535	I	PD-1<br>TIGIT	Drug: AB122,<br>Drug: AB154,<br>Drug: Placebo	Not yet recruiting
38	NCT04385173	I	B7-H3	Drug: B7-H3 CAR-T,<br>Drug: Temozolomide	Recruiting
39	NCT03576612	I	PD-1	Biological: AdV-tk,<br>Drug: Valacyclovir,<br>Radiation: Radiation,<br>Drug: Temozolomide,<br>Biological: Nivolumab,	Recruiting
40	NCT04047706	I	PD-1	Biological: IDO1 Inhibitor BMS-986205,<br>Biological: Nivolumab,<br>Radiation: Radiation Therapy,<br>Drug: Temozolomide	Recruiting
41	NCT02502708	I	IDO	Drug: Indoximod,<br>Drug: Temozolomide,<br>Radiation: Conformal Radiation,<br>Drug: Cyclophosphamide,<br>Drug: Etoposide	Completed
42	NCT02852655	I	PD-1	Drug: Pembrolizumab (MK-3475)	Recruiting
43	NCT03422094	I	PD-1<br>CTLA-4	Biological: NeoVax,<br>Biological: Nivolumab| Biological: Ipilimumab,<br>Procedure: Research blood draw, Procedure: Leukapheresis for research	Terminated
44	NCT02937844	I	PD-L1	Biological: Anti-PD-L1 CSR T cells, Drug: Cyclophosphamide,<br>Drug: Fludarabine	Unknown status
45	NCT03661723	II	PD-1	Drug: Pembrolizumab<br>Drug: Bevacizumab Radiation: Re-irradiation	Recruiting
46	NCT04118036	II	PD-1	Drug: Pembrolizumab<br>Drug: Abemaciclib	Suspended
47	NCT03722342	I	PD-1	Drug: TTAC-0001 and pembrolizumab combination	Active, not recruiting
48	NCT03405792	II	PD-1	Drug: Temozolomide (TMZ)<br>Device: Optune System<br>Drug: Pembrolizumab	Recruiting
49	NCT02530502	I	PD-1	Other: Laboratory Biomarker Analysis<br>Biological: Pembrolizumab<br>Radiation: Radiation Therapy<br>Drug: Temozolomide	Terminated
50	NCT02430363	I<br>II	PD-1	Drug: MK – 3475<br>Biological: Suppressor of the PI3K/Akt pathways	Unknown status
51	NCT03018288	II	PD-1	Drug: Pembrolizumab Biological: HSPPC-96<br>Drug: Temozolomide<br>Other: Placebo	Recruiting
52	NCT03347617	II	PD-1	Drug: Ferumoxytol<br>Other: Laboratory Biomarker Analysis<br>Procedure: Magnetic Resonance Imaging	Recruiting

				Biological: Pembrolizumab	
53	NCT03665545	I<br>II	PD-1	Drug: IMA950/Poly-ICLC and pembrolizumab	Recruiting
54	NCT04013672	II	PD-1	Drug: Pembrolizumab<br>Drug: SurVaxM<br>Drug: Sargramostim<br>Drug: Montanide ISA 51	Recruiting
55	NCT02337491	II	PD-1	Drug: Pembrolizumab<br>Drug: Bevacizumab	Completed
56	NCT02337686	II	PD-1	Other: Laboratory Biomarker Analysis<br>Biological: Pembrolizumab<br>Other: Pharmacological Study<br>Procedure: Therapeutic Conventional Surgery	Active, not recruiting
57	NCT03426891	I	PD-1	Drug: Pembrolizumab<br>Drug: Vorinostat<br>Drug: Temozolomide<br>Radiation: Radiotherapy	Recruiting
58	NCT03197506	II	PD-1	Radiation: External Beam Radiation Therapy<br>Other: Laboratory Biomarker Analysis<br>Biological: Pembrolizumab<br>Radiation: Radiation Therapy<br>Drug: Temozolomide<br>Procedure: Therapeutic Conventional Surgery	Recruiting
59	NCT02287428	I	PD-1	Radiation: Radiation Therapy<br>Biological: Personalized NeoAntigen Vaccine<br>Drug: Pembrolizumab<br>Drug: Temozolomide	Recruiting
60	NCT03311542		PD-1	Biological: pembrolizumab	No longer availabl e
61	NCT03797326	II	PD-1	Biological: Pembrolizumab<br>Drug: Lenvatinib	Recruiting
62	NCT01174121	II	PD-1	Biological: Young TIL<br>Drug: Aldesleukin<br>Drug: Cyclophosphamide<br>Drug: Fludarabine<br>Drug: Pembrolizumab (Keytruda)	Suspended
63	NCT04429542	I	PD-1	Drug: BCA101<br>Drug: Pembrolizumab	Recruiting
64	NCT03636477	I	PD-1	Biological: Ad-RTS-hIL-12<br>Drug: Veledimex<br>Drug: Nivolumab	Active, not recruiting
65	NCT02648633	I	PD-1	Radiation: Stereotactic Radiosurgery<br>Drug: Nivolumab<br>Drug: Valproate	Terminated
66	NCT03430791	II	CTLA-4, PD-1	Drug: Nivolumab 240 mg IV<br>Drug: Nivolumab 3 mg/kg<br>Drug: Ipilimumab 1 mg/kg<br>Device: NovoTTF200A (Optune)	Active, not recruiting
67	NCT03452579	II	PD-1	Drug: Nivolumab<br>Drug: Standard Dose Bevacizumab<br>Drug: Reduced Dose Bevacizumab	Active, not recruiting
68	NCT04195139	II	PD-1	Drug: Nivolumab<br>Drug: Temozolomide	Recruiting
69	NCT03743662	II	PD-1	Radiation: Re-irradiation (RT)	Recruiting

				Drug: Bevacizumab<br>Drug: Nivolumab<br>Procedure: Re-resection	
70	NCT03367715	II	CTLA-4, PD-1	Drug: Nivolumab<br>Drug: Ipilimumab<br>Radiation: Radiation Therapy (RT)	Recruiting
71	NCT02529072	I	PD-1	Drug: nivolumab<br>Biological: DC	Completed
72	NCT03718767	II	PD-1	Drug: Nivolumab	Recruiting
73	NCT02327078	I<br>II	PD-1	Drug: Nivolumab (Phase 1)<br>Drug: Epacadostat (Phase 1)<br>Drug: Chemotherapy (Phase 1)<br>Drug: Nivolumab (Phase 2)<br>Drug: Epacadostat (Phase 2)	Completed
74	NCT04323046	I	CTLA-4<br>PD-1	Biological: Ipilimumab<br>Biological: Nivolumab<br>Drug: Placebo Administration<br>Other: Quality-of-Life Assessment<br>Other: Questionnaire Administration	Recruiting
75	NCT02335918	I<br>II	PD-1	Drug: Combination of varlilumab and nivolumab	Completed
76	NCT03879512	I<br>II	CTLA-4<br>PD-1	Drug: depletion of regulatory T cells<br>Procedure: reoperation<br>Biological: cancer vaccine<br>Biological: checkpoint blockade Nivolumab/Ipilimumab	Recruiting
77	NCT03684811	I<br>II	PD-1	Drug: FT-2102<br>Drug: Azacitidine<br>Biological: Nivolumab<br>Drug: Gemcitabine and Cisplatin	Active, not recruiting
78	NCT04704154	II	PD-1	Drug: Regorafenib, (Stivarga, BAY73-4506)<br>Drug: Nivolumab (Opdivo)	Recruiting

Abbreviations: PD-1: programmed cell death protein 1, PD-L1: programmed death-ligand 1, CTLA-4: (Cytotoxic T-Lymphocyte Associated Protein 4, IDO: indoleamine 2,3-dioxygenase, Tim-3: T cell immunoglobulin and mucin-domain containing-3, TIGIT: T cell immunoreceptor with Ig and ITIM domain, LAG-3: lymphocyte activation gene-3, TNFRSF9: tumor necrosis factor receptor superfamily member 9

Supplementary Table 2: Completed clinical trials of oncolytic virotherapy for the treatment of GBM.

Virus	Strain	Features	Phase	Route	Trial Number
**Adenovirus**	Onyx-015	E1B-55kD deletion	I	-	-
	DNX-2401	Δ24 deletion in E1A RGD-4C fiber modification	I	IT	NCT00805376
	DNX-2401		I/II	Intracerebral infusion by CED	NCT01582516
	DNX-2401 + TMZ		I	IT followed by oral TMZ	NCT01956734 (Spain)
	DNX-2401 + IFNγ		Ib	IT followed by IFNγ	NCT02197169 (TARGET-I)
**Herpesvirus**	G207	γ34.5 and *UL39* deletion	Ib/II	IT	NCT00028158
			I	IT followed by radiation	NCT00157703
	HSV 1716	γ34.5 gene deletion	I	IT	(UK)
			I	IT	(UK)
			I	IT	(UK)
	G47Δ	γ34.5, *UL39* and α47 deletion. US11 expression under α47 promoter	I/II	IT	UMIN00000266
			II	IT	UMIN000015995 (Japan)
**Measles**	MV-CEA	Attenuated expressing hCEA	I	Injection into resection cavity and/or IT	NCT00390299
**Parvovirus**	ParvOryx	Unmodified rat parvovirus	I/IIa	IT/IV followed by tumor resection	NCT01301430
**Reovirus**	REOLYSIN	Unmodified human reovirus (serotype 3)	I	IT	-
			I/II	IT	NCT00528684
			I	IV	EudraCT 2011-005635-10
**Retrovirus**	Toca 511 +Toca FC	Expressing CD gene	I	Injection into resection cavity	NCT01470794
			I	IT or IV	NCT01156584
			I	IV	NCT01985256

Abbreviations: RGD: arginine-glycine-aspartic acid, CED: convection-enhanced delivery, IT: intratumorally, TMZ: temozolomide, IFN[illegible]: interferon gamma, CEA: carcinoembryonic antigen, IV: intravenously, CD: cytosine deaminase.

Supplementary Table 3: Currently active and/or recruiting clinical trials of oncolytic virotherapy for the treatment of GBM

Virus	Strain	Features	Phase	Route	Trial Number
**Adenovirus**	DNX-2401	Loaded onto allogeneic BM-hMSCs	I	IA	NCT03896568
	DNX2401+ pembrolizumab		II	IT	NCT02798406 (CAPTIVE)
	DNX-2440	DNX-2401 expressing OX40L	I	IT	NCT03714334
	NSC-CRAd-S-pk7	E1A under survivin promoter<br>Loaded onto NSCs	I	IT	NCT03072134
**Herpesvirus**	C134	γ34.5 deletion<br>IRS1 expression under control of an HCMV immediate early promoter	I	IT	NCT03657576
	G207<br>+/- 5 Gy radiation	Deletion of both copies of γ34.5 gene<br>Inactivating lacZ insertion in UL39 gene	I<br>I<br>II	IT<br>IT<br>IT	NCT02457845<br>NCT03911388<br>NCT04482933
	M032	Deletion of both copies of γ34.5 gene<br>Expressing human IL-12	I	IT	NCT02062827
	rQNestin34.5v2	γ34.5 and UL39 deletion<br>γ34.5 expression under nestin promoter	I	IT +/- IV<br>CPA	NCT03152318
**Poliovirus**	PVSRIPO	Attenuated poliovirus with IRES replaced by IRES from HRV2	I/Ib<br>Ib<br>II	IT<br>IT<br>IT	NCT01491893<br>NCT03043391<br>NCT02986178
	PVSRIPO+ pembrolizumab		II	IT	NCT04479241 (LUMINOS-101)
**Reovirus**	REOLYSIN<br>+ rGM-CSF	Unmodified human reovirus (serotype 3)	I	IV	NCT02444546
**Retrovirus**	Toca 511 +Toca FC	Expressing CD gene	II/III	IT	NCT02414165
**Vaccinia**	TG6002<br>+ 5-FC	Expressing FCU1<br>TK and RR gene deletion	I/II	IV	NCT03294486

Abbreviations: BM-hMSCs: bone-marrow human mesenchymal stem cells, IA: intra-arterial, IT: intratumorally, OX40L: OX40 ligand, NSCs: neural stem cells, IRS1: insulin receptor substrate 1, HCMV: human cytomegalovirus, UL39: ribonucleotide reductase large subunit, IL-12: interleukin 12, IV: intravenously, CPA: cyclophosphamide, IRES: internal ribosome entry site, HRV2: human rhinovirus type 2, rGM-CSF: recombinant granulocyte-macrophage colony-stimulating factor, CD: cytosine deaminase, 5-FC: 5-fluorocytosine, FCU1: fusion suicide gene 1, TK: thymidine kinase, RR: ribonucleotide reductase.

Supplementary Table 4: Currently active and/or recruiting clinical trials of CAR-T cell therapy for the treatment of GBM

	Target Antigen	Estimated Enrolment	Status	Phase	Trial Number
**1**	GD2		Withdrawn	II	NCT03252171
**2**	EphA2		Withdrawn	II	NCT02575261
**3**	EGFR		Unknown	I	NCT02331693
**4**	GD2		Withdrawn	II	NCT04406610
**5**	EGFRVIII, IL13Rα2, Her-2, EphA2, CD133, GD2	100 participants	Recruiting	I	NCT03423992
**6**	GD2	54 participants	Recruiting	I	NCT04196413
**7**	GD2-C7R	34 participants	Recruiting	I	NCT04099797
**8**	B7-H3	70 participants	Recruiting	I	NCT04185038
**9**	MUC1	20 participants	Unknown	II	NCT02617134
**10**	CD147	31 participants	Recruiting	I	NCT04045847
**11**	Chlorotoxin-Car T cells for MPP2$^+$ Glioblastoma cells	36 participants	Recruiting	I	NCT04214392
**12**	EGFRvIII	18 participants	Completed	II	NCT01454596
**13**	EGFRvIII	3 participants	Terminated	I	NCT02664363
**14**	NKG2D	10 participants	Not yet recruiting	I	NCT04550663
**15**	EGFR	36 participants	Recruiting	I	NCT03638167
**16**	HER2	48 participants	Recruiting	I	NCT03500991
**17**	EGFRvIII, DR5, NY-ESO-1, Mesothelin	50 participants	Recruiting	II	NCT03941626
**18**	HER2		Withdrawn	II	NCT02713984
**19**	EGFRvIII	73 participants	Recruiting	II	NCT03638206
**20**	EGFRvIII	11 participants	Terminated	I	NCT02209376
**21**	IL13Rα2	92 participants	Recruiting	I	NCT02208362
**22**	EGFRvIII	24 participants	Suspended	I	NCT03283631
**23**	EGFRvIII	20 participants	Unknown	I	NCT02844062
**24**	NKG2D		Withdrawn	I	NCT04270461
**25**	HER2	42 participants	Recruiting	I	NCT03389230
**26**	IL13Rα2	30 participants	Recruiting	I	NCT04661384
**27**	IL13Rα2	60 participants	Recruiting	I	NCT04003649
**28**	EGFRvIII	7 participants	Completed	I	NCT03726515
**29**	PD-L1	20 participants	Unknown	I	NCT02937844
**30**	NKG2D	20 participants	Not yet recruiting		NCT0471799
**31**	B7-H3	40 participants	Recruiting	II	NCT04077866
**32**	B7-H3	12 participants	Recruiting	I	NCT04385173
**33**	HER2	16 participants	Completed	I	NCT01109095

Abbreviations: GD2: disialoganglioside, EphA2: erythropoietin-producing hepatocellular carcinoma A2, CLTX: chlorotoxin, NKG2DLs: natural killer group 2D ligands, CSPG4: chondroitin sulfate proteoglycan 4, CAIX: carbonic anhydrase IX, and integrin αvβ3, EGFR: epidermal growth factor receptor, EGFRVIII: EGFR variant III, IL13Rα2: interleukin-13 receptor subunit alpha-2, Her-2: human epidermal growth factor receptor 2, MUC1: mucin 1, MMP2: multipotent progenitor 2, DR5: death receptor 5, NY-ESO-1: New York esophageal squamous cell carcinoma 1, PD-L1: **p**rogrammed death-ligand 1
